# Characterization of multiple sequence alignment errors using complete-likelihood score and position-shift map

**DOI:** 10.1186/s12859-016-0945-5

**Published:** 2016-03-18

**Authors:** Kiyoshi Ezawa

**Affiliations:** Department of Bioscience and Bioinformatics, Kyushu Institute of Technology, Iizuka, 820-8502 Japan; Department of Biology and Biochemistry, University of Houston, Houston, TX 77204-5001 USA

**Keywords:** Multiple sequence alignment (MSA), Error, Visualization, Likelihood, Stochastic evolutionary model, Insertion/deletion (indel), Power-law, Probability distribution, MSA space exploration

## Abstract

**Background:**

Reconstruction of multiple sequence alignments (MSAs) is a crucial step in most homology-based sequence analyses, which constitute an integral part of computational biology. To improve the accuracy of this crucial step, it is essential to better characterize errors that state-of-the-art aligners typically make. For this purpose, we here introduce two tools: the complete-likelihood score and the position-shift map.

**Results:**

The logarithm of the total probability of a MSA under a stochastic model of sequence evolution along a time axis via substitutions, insertions and deletions (called the “complete-likelihood score” here) can serve as an ideal score of the MSA. A position-shift map, which maps the difference in each residue’s position between two MSAs onto one of them, can clearly visualize where and how MSA errors occurred and help disentangle composite errors. To characterize MSA errors using these tools, we constructed three sets of simulated MSAs of selectively neutral mammalian DNA sequences, with small, moderate and large divergences, under a stochastic evolutionary model with an empirically common power-law insertion/deletion length distribution. Then, we reconstructed MSAs using MAFFT and Prank as representative state-of-the-art single-optimum-search aligners. About 40–99% of the hundreds of thousands of gapped segments were involved in alignment errors. In a substantial fraction, from about 1/4 to over 3/4, of erroneously reconstructed segments, reconstructed MSAs by each aligner showed complete-likelihood scores not lower than those of the true MSAs. Out of the remaining errors, a majority by an iterative option of MAFFT showed discrepancies between the aligner-specific score and the complete-likelihood score, and a majority by Prank seemed due to inadequate exploration of the MSA space. Analyses by position-shift maps indicated that true MSAs are in considerable neighborhoods of reconstructed MSAs in about 80–99% of the erroneous segments for small and moderate divergences, but in only a minority for large divergences.

**Conclusions:**

The results of this study suggest that measures to further improve the accuracy of reconstructed MSAs would substantially differ depending on the types of aligners. They also re-emphasize the importance of obtaining a probability distribution of fairly likely MSAs, instead of just searching for a single optimum MSA.

**Electronic supplementary material:**

The online version of this article (doi:10.1186/s12859-016-0945-5) contains supplementary material, which is available to authorized users.

## Background

The reconstruction of multiple sequence alignments (MSAs) is a crucial step in most of the advanced homology-based sequence analyses, because it prepares input data fed into a wide variety of bio-macromolecular analyses in computational biology, such as the inference of phylogenetic relationships among DNA/RNA/protein sequences [1, 2], the prediction of their 3D structures [3], the prediction and comparison of their functions [4–6], and the identification of their sites or regions under selection [7]. Because of such importance, it is essential to reconstruct MSAs as accurately as possible. The development of MSA aligners, i.e., programs to reconstruct MSAs, has a long history (see, e.g., [8–10]). Since the advent of CLUSTALW about two decades ago [11], it had been a de facto standard for a long time. Then, it was followed by the “next generation” of aligners, such as T-Coffee [12], MAFFT [13–15], Muscle [16, 17], ProbCons [18], Prank [19, 20], MUMMALS [21], ProbAlign [22], M-Coffee [23] and DIALIGN-TX [24], which feature improved computational speed and/or accuracy, and currently they and the upgraded CLUSTALW can be regarded as the state-of-the-art programs to search for an optimum MSA. Nevertheless, even recently, the accuracy of reconstructed MSAs has frequently come into question (e.g., [20, 25–28]). For example, Wong et al. [26] pointed out that different aligners produce different MSAs, which often result in conflicting conclusions on the sequence phylogeny or positively selected sites, and they hypothesized that uncertainty in the alignment can lead to such problems. Landan and Graur [27] estimated that, depending on the sequence divergences, about 5–90% of the “homologous” residue pairs in each reconstructed MSA are erroneous. MSA errors influence the results, and even the conclusions, of the downstream analyses (e.g., [26, 29–33]). Especially, MSA errors impact the inference of insertions/deletions (indels) directly [20, 28], because the MSA reconstruction problem is, after all, how to place gaps, and because gaps are usually supposed to represent the effects of indels on the sequences. Thus, the success of methods to infer the history of indels from a given MSA (e.g., [34, 35]) depends heavily on how accurately the MSA was reconstructed.

The exact purpose of this study is to gain new insights into how to further improve the accuracy of MSA reconstruction. For this purpose, we will look into MSA errors from angles different from those attempted thus far. We consider that there are at least three major causes of MSA errors: (i) discrepancies between the score and the true likelihood of a MSA, (ii) inadequate exploration of the MSA space, and (iii) the stochastic nature of sequence evolutionary processes. First, any MSA scoring system must have been devised so that the MSA with the highest score will be the most likely under some “model” of MSA creation, which may have been conceived explicitly or implicitly. As the “model” represents (the outcomes of) the natural evolutionary processes more faithfully, the MSA score will approximate the true occurrence probability, or the “true likelihood,” of a MSA more accurately. In reality, however, the highest scoring of a MSA under a certain scoring system does not necessarily guarantee that the MSA is truly most likely to be generated through real evolutionary processes. This is conspicuous for “similarity-based” aligners that use some sum-of-pairs scoring systems or their derivatives (as classified by Blackburne and Whelan in [36]), as they do not faithfully reflect evolutionary processes, especially those of insertions/deletions (indels) (see, e.g., [20]). But, although subtle, “evolution-based” aligners (also as classified in [36]) are not completely immune to this problem, either, because their scores are usually based on probabilistic models that are not genuine stochastic evolutionary models of realistic sequence evolution. Here a “genuine stochastic evolutionary model,” or an “evolutionary model” for short, means a stochastic model that describes the evolution of an entire sequence along a time axis (and thus down a phylogenetic tree) via substitutions and indels without unnatural restrictions on the processes. (For more details, see, e.g., the “Background” section of [37].) Second, it is inevitable that a practical MSA aligner explores only a portion of the entire MSA space. The dynamic programming (DP) for pairwise alignment (PWA) reconstruction (e.g., [38–40]) can explore the entire PWA space with the time complexity from *O*(*L*^2^) to *O*(*L*^3^), where *L* denotes the dimension of the sequence length. However, applying DP to the MSA search would take at least *O*(*L*^*k*^) (*k* is the number of sequences) or practically forever for a set of sequences commonly analyzed (e.g., [41]; Section 14.6 of [8]). Thus, inevitably, all current practical MSA aligners resort to heuristics. The commonest among them are the progressive alignment (e.g., [42]; Section 14.10 of [8]) and the iterative MSA refinement [43, 44], both of which are techniques that repeatedly apply the pairwise DP to align, or re-align, two sub-MSAs. Although these techniques enable the alignment to finish within a reasonable amount of time, they could leave some important regions of the MSA space unexplored. And third, it should be kept in mind that the evolutionary process on a sequence is stochastic by nature (e.g., [45, 46]). Thus, even if we were equipped with an ideal scoring system under which the truly most likely MSA always scores the highest, and even if we could somehow explore the entire MSA space, the optimum MSA thus identified could still be different from the true MSA that resulted from the actual evolutionary process. In order to contemplate strategies to improve the MSA accuracy, it is therefore important to figure out the relative frequencies of MSA errors by these three causes. To this end, we here use the “complete-likelihood score” of a MSA, which is the logarithm of the total probability that the MSA resulted under an evolutionary model (see section *R1* of *Results and discussion* and section *M5* of *Methods*), as a good proxy for the true log-likelihood of the MSA.

Another essential issue is the number of moves by which the true MSA is separated from the reconstructed MSA. If the true MSA is quite “close” to the reconstructed MSA in a majority of MSA errors, the strategy to explore only the vicinity of the reconstructed MSA could be practical. If, on the other hand, the true MSA is mostly “far away” from the reconstructed MSA, we may need to come up with a totally new strategy to search for the true MSA. To address this issue, we introduce another new tool, the “position-shift map” (see section *R1* of *Results and discussion* and section *M6* of *Methods*), which can directly estimate the number of moves separating the two MSAs. The tool will also help examine MSA errors at the single-residue level, because it focuses directly on the gap misplacement itself.

We chose to examine MSA errors by two state-of-the-art aligners, MAFFT [13–15] and Prank [19, 20], as representatives of similarity-based and evolution-based aligners [36], respectively. Although there are many other state-of-the-art aligners searching for a single optimum, such as the aforementioned ClustalW, T-Coffee, Muscle, ProbCons, ProbAlign, DIALIGN-TX, MUMMALS, all aligners listed here were classified as similarity-based in [36]. And other state-of-the-art aligners should also be classified into either of the two types. Thus, we expect that the characteristics of errors by MAFFT and Prank should well represent errors by other similarity-based aligners and evolution-based aligners, respectively.

In order to identify errors in each reconstructed MSA, we need to compare it with the true MSA of the identical sequence set. Traditional evaluations of the MSA accuracy and characterizations of MSA errors used some benchmark sets of reference MSAs, typically based on structural alignments, such as HOMSTRAD [47], BAliBASE [48] PREFAB [17], SAD [49], and SABmark [50]. An inevitable problem in principle with these benchmark sets is that nobody knows whether or not each reference MSA is indeed the true MSA that faithfully reflects the actual evolutionary process. Unfortunately, this is unavoidable, even though structural alignments may in general be more accurate than exclusively sequence-based alignments, or even though dedicated experts may have manually edited the reference MSAs to enhance their credibility. Besides, when studying, e.g., the evolution or functions of non-coding DNA sequences (e.g., [51]), we cannot usually rely on the 3D structural information. Because this study crucially depends on the true MSA in order to identify and characterize MSA errors precisely, we chose to use a set of reference MSAs generated by computer simulations via Dawg [52]. Dawg was shown to satisfy some critical criteria for a genuine simulator of the molecular evolution of DNA and protein sequences [53]. Moreover, it also enables us to use the biologically more realistic Zipf power-law distributions of indel lengths (e.g., [54] and references therein). These features of Dawg guarantee that each reference MSA was indeed generated through a somewhat realistic evolutionary process.

In this study, we attempt to characterize MSA errors by similarity-based and evolution-based aligners, by using the complete-likelihood score and the position-shift map. We would like to stress that our purpose here is to characterize MSA errors in order to find clues for improving MSA accuracy but *not* to compare the accuracy of the aligners. Those who are interested in the latter should read the aforementioned references on the aligners and the benchmark MSA sets. In *Results and discussion*, after briefly explaining these two tools (in section *R1*), we discuss the results of the analyses using them. In its last section (*R7*), the scope of this study is discussed. In *Methods*, we describe how we did the analyses, including how we prepared the input data. Some details on the analyses and the underlying theories are described in *Supplementary methods* in Additional file 1.

## Results and discussion

### R1. New tools: complete-likelihood score and position-shift map

The complete-likelihood score is the log-likelihood, under a given MSA, of a genuine stochastic evolutionary model. By definition, the MSA with the highest complete-likelihood score should have most probably resulted from actual evolutionary processes, provided that the evolutionary model faithfully represents the processes. Although similar notions (such as the “marginal probability of a MSA”) and their program implementations were proposed in the past (e.g., [28, 55–59]), these studies harbor at least one of two problems. One problem is that they were based on probabilistic models that are not evolutionary models. (For more details on this issue, see, e.g., “Background” of [37] and section 2 of [60].) The other problem is that their models inevitably depended on geometric distributions of indel lengths, which are substantially different from the empirically established power-law distributions (e.g., [54] and references therein). In a previous study [61], we proposed an algorithm to quite accurately calculate the occurrence probability of a MSA under an evolutionary model of indels with any length distributions including power-laws. By combining this algorithm with an existing algorithm or program that calculates the occurrence probability of a MSA through substitution processes (e.g., [1, 2, 7, 62, 63]), we should be able to calculate the complete-likelihood score of a MSA exactly under the evolutionary model used by a genuine sequence evolution simulator as presented, e.g., in [52, 53, 64] (section *M5* of *Methods*). One of the key results in this study is the proof that the complete likelihood score can be calculated as the summation of the substitution component and the indel component, given some commonly satisfied conditions. This result substantially facilitates the calculation of the complete likelihood, once we know how to calculate its indel and substitution components. The proof is somewhat long and thus given in section *SM-1* of *Supplementary methods* in Additional file 1. 1

**Fig. 1:**
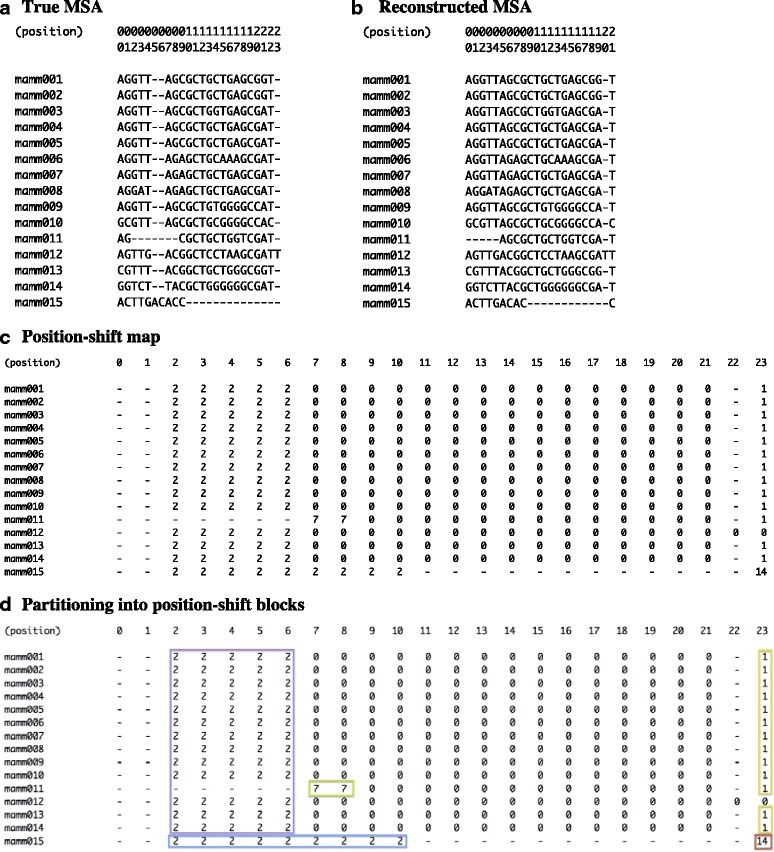
Example position-shift map. **a** A true MSA, which was created by a simulation along the tree in Fig. 2b. **b** A reconstructed MSA. In the position-shift map (**c**), each site of each sequence is occupied by the residue’s horizontal position in the reconstructed MSA minus that in the true MSA. **d** Partitioning the map into position-shift blocks (enclosed by *colored boxes*). Each of the *yellow* and *green* blocks (with shifts 7 and 1, respectively,) was associated with a “shift.” The *blue* block (with shift 2) and the *red* one (with shift 14) were paired and associated with a “merge + shift.” The *purple* one (with shift 2) was judged as accompanying the *blue* one to result in the “merge.” [NOTE: The *rectangles* in *panel* **d** were drawn manually, based on the output of a prototype script to parse a position-shift map.]

**Fig. 2:**
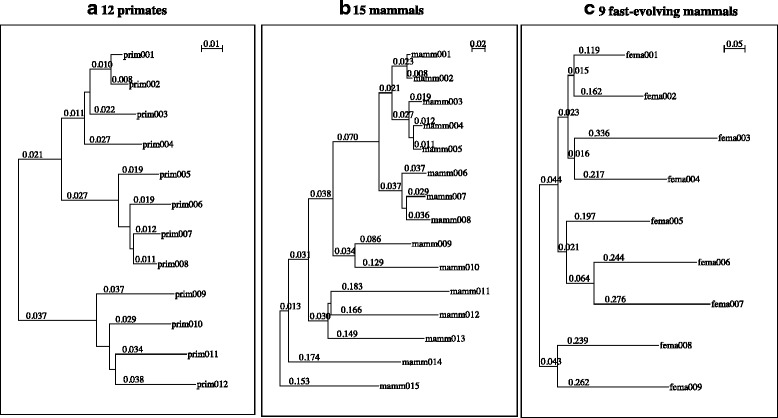
Phylogenetic trees used for simulated DNA sequence evolution. **a** The tree of 12 primates. **b** The tree of 15 mammals. **c** The tree of 9 fast-evolving mammals. The *number* along each branch is its length (in the expected number of substitutions per 4-fold degenerate site). Additional file 1: Table S1 associates the sequence IDs with species names

The other tool, the position-shift map, helps visualize how two MSAs of an identical sequence set differ from each other (Fig. 1; section *M6* of *Methods*). The “position-shift” of each residue (base or amino-acid) is its horizontal position along the reconstructed MSA minus that along the true MSA, and a position-shift map is created from one of the two MSAs by replacing the residues in it with the corresponding position-shifts. The map clarifies differences between two MSAs (e.g., Fig. 1, panels a and b) at a residue-level resolution, by focusing directly on the gap misplacement itself (Fig. 1c). In contrast, traditionally used measures of MSA accuracy, such as the sum-of-pairs score and the column score [9], are based on the proportion of correctly inferred homologous residue relationships. Given two MSAs of the same homologous sequences, the construction of the position-shift map is unique, simple, and quick (basically requiring just a single read through of the two MSAs). And the map itself should be very useful for manual inspections of MSA errors, as exemplified by panel c of Fig. 1. For massive analyses, however, we need to computerize the extraction of information from the position-shift map. For this purpose, we introduce “position-shift blocks,” each of which consists of contiguous residues with the same position-shift (panel d). A position-shift map is partitioned into blocks with the help of the phylogenetic tree of aligned sequences (Fig. 2b; section *M6* of *Methods*). The block can define a single step of move from one MSA to the other. Therefore, we can also use the map to estimate “how far” the two MSAs are separated from each other, and possibly to disentangle a composite MSA error into a set of “elementary” errors. (See also Additional file 1: Figures S1, S2 and S3 for other examples.)

### R2. Overall statistics on simulated and reconstructed MSAs

We simulated MSAs using Dawg [52], with a Zipf power-law distribution of indel lengths as empirically established (see *Background*). Each simulation started with a 1000-base DNA sequence at the root. Other parameters were chosen to be typical of the neutral evolution of mammalian DNA sequences [65]. More precisely, we created three MSA sets: the first along the phylogenetic tree of 12 primates (Fig. 2a), the second along the tree of 15 mammals (Fig. 2b), and the third along the tree of 9 fast-evolving mammals (Fig. 2c). These three sets were intended to represent homologous DNA sequences with small, moderate, and large divergences, respectively (Table 1; see Additional file 1: Table S1 for species names). See section *M1* of *Methods* for details on the simulations. In total, the simulated MSASs in these three sets consisted of 403,394, 966,017 and 309,425 gapped segments, respectively, as well as gapless segments separating them (Table 2).

**Table 1:** Representative branch lengths for 3 phylogenetic trees used for simulations

Phylogenetic tree	12 primates	15 mammals	9 fast-evolving mammals
Total branch length ^a^	0.384	1.539	2.279
All branches	0.017 ± 0.012	0.055 ± 0.059	0.142 ± 0.111
External branches	0.022 ± 0.011	0.080 ± 0.070	0.228 ± 0.064
Internal branches	0.012 ± 0.012	0.026 ± 0.018	0.032 ± 0.018

NOTE: Each branch length is measured in terms of the expected number of substitutions per base. Except in the first row, the numbers in each cell are: average ± standard deviation.

^a^The summation of branch lengths over all branches in the tree.

**Table 2:** Basic statistics on simulated (i.e., true) MSAs and reconstructed MSAs

		Dataset		
Subjects counted	Aligner ^a^	12 primates	15 mammls	9 fast-evolving mammals
[True MSAs]				
1. MSAs	—	10,000	10,000	3000
2. Gapped segments	—	404,394	966,017	309,425
3. Gapped segments with long indels ^b^ (%) ^c^	—	446 (0.1%)	9503 (1.0%)	5784 (1.9%)
[Reconstructed MSAs]				
4. All erroneous segments	MAFFT-1 ^a^	145,002	320,455	29,781
	MAFFT-i ^a^	139,701	352,482	58,372
	Prank	135,602	374,087	39,315
5. True gapped segments in item 4 (%) ^c^	MAFFT-1	182,712 (45.2%)	836,766 (86.6%)	305,756 (98.8%)
	MAFFT-i	173,701 (43.0%)	813,591 (84.2%)	300,879 (97.2%)
	Prank	150,618 (37.2%)	722,767 (74.8%)	300,359 (97.1%)
6. Erroneous segments without long indels ^d^	MAFFT-1	144,422	308,923	24,239
	MAFFT-i	139,144	340,865	51,912
	Prank	135,097	363,961	34,907
7. True gapped segments in item 6 (%) ^c^	MAFFT-1	181,868 (45.0%)	781,784 (80.9%)	171,008 (55.3%)
	MAFFT-i	172,915 (42.8%)	772,818 (80.0%)	234,311 (75.7%)
	Prank	150,010 (37.1%)	676,950 (70.1%)	175,967 (56.9%)

^a^The aligner labels, “MAFT-1” and “MAFFT-i” stand for E-INS-1 (a progressive mode) and E-INS-i (an accuracy-oriented iterative mode), respectively, of MAFFT

^b^Each of these segments involves at least one apparent indel longer than 100 bases

^c^These percentages are relative to the number of all true gapped segments (in item 2) in the same column

^d^In these segments, neither the true MSAs nor the reconstructed MSAs involve any apparent indels longer than 100 bases each

Then, after removing all gaps from the simulated MSAs, the MSAs were reconstructed using MAFFT [15] and Prank [20] (see section *M2* of *Methods*). For MAFFT, we used an accuracy-oriented option, E-INS-i, because it was intended to perform well on MSAs with long gaps, which are expected to be quite common in our simulated MSA sets. To examine the effect of the iterative refinement, we also performed E-INS-1 of MAFFT, which is a progressive-only option and is equivalent to E-INS-i with no iterative refinement. For Prank, we tried two sets of parameters, one default and the other “best-fit.” Because MSAs with the “best-fit” setting seemed slightly more accurate and slightly more stable against perturbations of simulation conditions in our preliminary analysis (data not shown), we will henceforth discuss only the results with this setting.

After pre-processing (section *M3* of *Methods*, and section *SM-2* of *Supplementary methods* in Additional file 1), each simulated true MSA and its reconstructed counterpart were compared and the pair of MSAs was chopped into an alternating series of correctly aligned segments (“correct segments” for short) and erroneously aligned segments (“erroneous segments” for short) as in [27] (see section *M4* of *Methods*, and section *SM-3* of *Supplementary methods* in Additional file 1 for details). The basic statistics on the reconstructed MSAs are also shown in Table 2. Briefly, about 37–45%, 75–87% and 97–99% of true gapped segments were involved in the errors of MSAs among 12 primates, 15 mammals, and 9 fast-evolving mammals, respectively, regardless of the alignment methods (item 5 of the table). In order to avoid extremely long computations, the analyses described hereafter were performed after removing erroneous segments containing apparent indels that are over 100 bases long. The fractions of the true gapped segments lost by this screening were small for 12 primates and 15 mammals, but they were substantial for 9 fast-evolving mammals (compare items 5 and 7 of the table). Therefore, it should be kept in mind that the actual situations with large sequence divergences could be severer than what the data presented below will indicate.

### R3. Comparisons of complete-likelihood scores and of aligner-specific scores

**Fig. 3:**
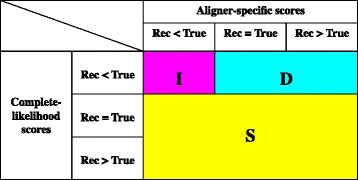
Definitions of three broad score categories, “D,” “I” and “S.” The “<,” “=“and “>” represent the results of the comparisons of the scores of the two MSAs. The “Rec” and “True” stand for the score of the reconstructed MSA and that of the true MSA, respectively. See the text for the rationale underlying these definitions of the categories

As argued in *Background*, we consider that there are at least three major causes of MSA errors: (i) discrepancies between the score and the true likelihood of a MSA, (ii) inadequate MSA space exploration, and (iii) the stochastic nature of evolutionary processes. By comparing the reconstructed MSA with the true MSA in each erroneous segment in terms of both the complete-likelihood score and the “aligner-specific score,” which was calculated according to the aligner’s own scoring scheme (section *M5* of *Methods*), we classified the erroneous segments into three broad score categories. We named them “D,” “I” and “S,” taking the first letters of the above three causes. Their definitions are graphically shown in Fig. 3. The rationale for these definitions is as follows. First, if the true MSA does not have a larger complete-likelihood score than the reconstructed MSA, we cannot identify the true MSA even via an ideal single-optimum search for the truly most likely MSA. Therefore, such erroneous segments are categorized as “S.” In each remaining segment, the true MSA has a larger complete-likelihood score than the reconstructed MSA, and thus it may be identified via an ideal single-optimum search. Especially, if the true MSA has a larger aligner-specific score than the reconstructed MSA, an improved MSA space exploration alone may suffice for the reconstruction of the true MSA. Thus, such cases are categorized as “I.” The rest exhibits discrepancies between the comparison of aligner-specific scores and that of complete-likelihood scores. Thus, it is classified as “D.” It should be kept in mind, however, that a better score of the true MSA than that of the reconstructed MSA does not necessarily mean that the true MSA has the best score, whereas a lower-score of the true MSA than that of the reconstructed MSA always means that the true MSA is not best scoring. This fact suggests that the proportions of “S” and “I” reported below should be lower bounds and upper bounds, respectively.

**Fig. 4:**
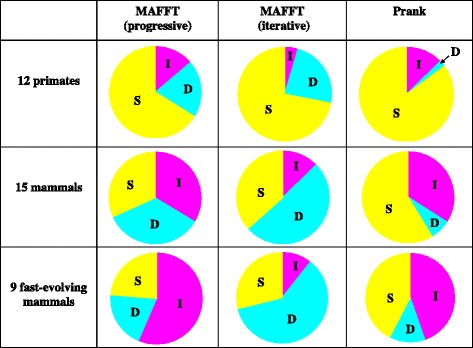
Proportions of three broad score categories. Each *pie chart* shows the proportions of 3 broad score categories, I (*magenta*), D (*cyan*) and S (*yellow*), in a particular MSA set (*row*) via a specified alignment method (*column*). The specific “progressive” and “iterative” options of MAFFT are E-INS-1 and E-INS-i, respectively. For the definitions of the 3 categories, see Fig. 3. For numerical values of the proportions and the absolute frequencies, see Additional file 1: Table S2

The results of this analysis are summarized in Fig. 4 (The numerical data are provided in Additional file 1: Table S2). First, we notice that category “S” always accounts for a substantial fraction, from approximately 1/4 to more than 3/4, indicating the huge impact of the stochastic nature of evolutionary processes. The impact may be bigger than what the figure naively indicates, because of the caveat in the previous paragraph. Second, we notice that the relative influences of categories “I” and “D” differ greatly between MAFFT (iterative) and Prank; “D” predominates in errors via MAFFT and “I” predominates in errors via Prank. The latter indicates that the scoring scheme of Prank generally does a better job in identifying truly most likely MSAs,^2^ but that its MSA space exploration (via progressive alignment) is not enough. In contrast, the former indicates that the scoring scheme of MAFFT does not represent the true likelihoods of MSAs so faithfully, but that its iterative refinement strategy can search for optimum MSAs (in terms of the aligner-specific score) quite efficiently. The efficiency of the iterative refinement strategy was also corroborated by the comparison between the progressive and the iterative options of MAFFT (Fig. 4). Another interesting observation would be that the proportion of “I” via MAFFT (progressive) is nearly equal to that via Prank regardless of the sequence divergence. This may not be a coincidence, because both resort to the progressive alignment for the MSA space exploration.

### R4. Position-shift map analysis (i): separation between true and reconstructed MSAs

Here, we examine “how far” the reconstructed MSA is separated from the true MSA in each erroneous segment, using the position-shift map (like Fig. 1 and Additional file 1: Figures S1, S2 and S3). As briefly explained in section *R1* and section *M6* of *Methods*, the position-shifts mapped onto a MSA are lumped into several blocks (panel d of aforementioned figures). (For details, see section *SM-4* of *Supplementary methods* in Additional file 1.) In principle, the number of such blocks (excluding the background block(s)) should represent the number of topological steps (or “block-wise moves”) necessary to transform the reconstructed MSA into the true MSA. Another important measure is the “total path length” between the two MSAs, which represents the number of sites whose positions need to be switched (in subsets of sequences) until the two MSAs match. Thus it is the number of “site-wise moves.” We approximated it by the summation of the lengths of the non-background position-shift blocks in each erroneous segment. We also tallied the sizes of the position-shift blocks.

Additional file 1: Figures S4 and S5 show their distributions for MAFFT (iterative), Prank and MAFFT (progressive). On the same MSA set, the distributions are quite similar among different alignment methods, and the distributions get broader and broader as the sequence divergences increase. What attracts our particular interests is the proportion of erroneous segments in which the reconstructed MSA is close to the true MSA. Considering the feasibility of a simple exploration strategy by multiple block-shifts, we regarded the two MSAs as “close” to each other if they are separated only by less than 5 block-wise moves, or less than 30 site-wise moves. (Incidentally, the average size of an erroneous segment was about 30 sites or more for 15 mammals.) Table 3 and Additional file 1: Table S3 show the complements, i.e., the proportions of the erroneous segments where the true and reconstructed MSAs are “far apart” from each other. Overall, regardless of the alignment method, when the sequence divergences are small or moderate, the two MSAs are “close” to each other in a majority of the erroneous segments: about 99% or more for 12 primates and about 80% for 15 mammals. For 9 fast-evolving mammals, however, the two MSAs are “close” to each other in only about a half of the segments. Remember that intractable segments, which account for a substantial fraction in this data set, were excluded from the analysis. Thus, the segments in which the two MSAs are “close” to each other should actually be a minority for large sequence divergences.

**Table 3:** Erroneous segments in which reconstructed MSA is “far-apart” from true MSA

	w/ many block-wise steps ^a^		w/ many site-wise steps ^b^		long blocks ^c^	
Score category	MAFFT (E-INS-i)	Prank (Best-fit)	MAFFT (E-INS-i)	Prank (Best-fit)	MAFFT (E-INS-i)	Prank (Best-fit)
12 primates						
I	0.6%	1.7%	1.4%	1.8%	0.6%	0.9%
D	2.0%	6.8%	4.1%	8.6%	1.7%	3.1%
S	0.1%	0.1%	0.1%	0.2%	0.1%	0.1%
Overall	0.5%	0.4%	1.1%	0.6%	0.6%	0.4%
15 mammals						
I	18.8%	27.2%	16.8%	22.5%	3.0%	5.7%
D	36.1%	54.1%	33.6%	49.3%	5.8%	8.0%
S	4.4%	7.7%	3.2%	5.3%	1.0%	1.6%
Overall	22.3%	17.6%	20.3%	14.3%	4.7%	4.6%
9 fast-evolving mammals						
I	40.1%	51.5%	36.9%	47.7%	4.1%	7.1%
D	68.9%	83.4%	66.5%	80.9%	7.1%	7.8%
S	16.6%	24.1%	13.9%	19.6%	2.7%	2.9%
Overall	50.7%	43.9%	48.2%	40.0%	6.5%	6.7%

NOTE: Shown in each cell is the percentage of each specified score category (row) that the errors with each specified type of “apart”-ness (column) account for

^a^ The true and reconstructed MSAs are separated by 5 or more block-wise steps

^b^ The true and reconstructed MSAs are separated by 30 or more site-wise steps

^c^ Position-shift blocks that are 30 or more sites long

The tables also indicate that the proportions differ considerably among the 3 broad score categories. In short, category “S” contains larger proportions of segments in which the two MSAs are “close” to each other, whereas category “D” contains smaller proportions of such segments. This result has an important implication for possible strategies to improve the MSA accuracy (section *R6*).

### R5. Position-shift map analysis (ii): tallying MSA errors of different types

**Fig. 5:**
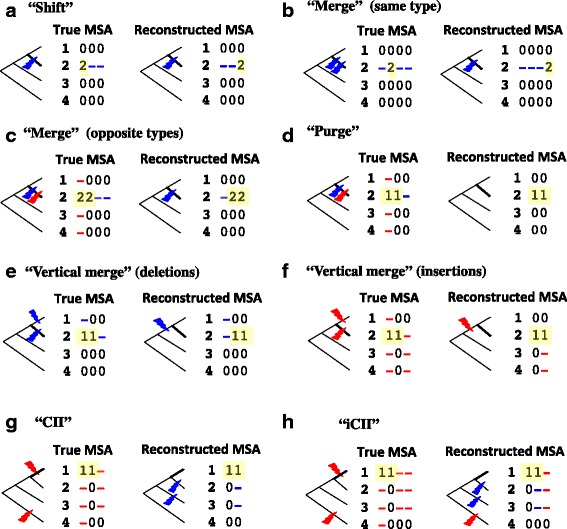
“Elementary” MSA errors associated with single position-shift blocks. The figure schematically illustrates a “shift” [(**a**)], a “merge” of the events of the same type [(**b**)], a “merge” of the events of opposite types [(**c**)], a “purge” [(**d**)], a “vertical merge” of two deletions [(**e**)], a “vertical merge” of two insertions [(**f**)], a “collapse of independent insertions (CII)” [(**g**)], and an “incomplete collapse of independent insertions (iCII)” [(**h**)]. In each *panel*, the tree and the position-shift map on the *left* are for the true MSA, and those on the *right* are for the reconstructed MSA. In each position-shift map, the position-shift block is highlighted in *yellow*, a *red* gap was derived from an (spurious) insertion, and a *blue* gap was derived from a (spurious) deletion. On each tree, the *thick branch* delimits the position-shift block, and a *red lightning bolt* and a *blue lightning bolt* represent an insertion and a deletion, respectively, any of which may be spurious

In order to explore the MSA space starting from a reconstructed MSA, it would be useful to learn what types of MSA errors are common. Landan and Graur [27] classified pairwise alignment (PWA) errors into five types: a “shift,” which is a re-positioning of a single gap not influencing any neighboring ones; a “merge” of two neighboring gaps, resulting in a single incorrect gap; a “purge” of a pair of gaps with the same size and on opposite sequences; a “split” of a gap into two incorrect gaps; an “ex-nihilo,” which creates a pair of spurious gaps of the same size on opposite sequences out of a gapless region.^3^ (A “split” is the reverse of a “merge,” and an “ex-nihilo” is the reverse of a “purge.”) And errors that cannot be classified as any of the above were tentatively put into the “complex” category. These five types apply also to MSA errors, if we re-interpret a MSA as a PWA of two complementary sub-MSAs (Fig. 5, panels a-d). Furthermore, because of an additional temporal (or phylogenetic) dimension, some other types of MSA errors can also be defined. For example, we here define a “vertical merge” (and a “vertical split” as its reverse), a “collapse of independent insertions (CII)” (and a “creation of spurious independent insertions (CSII)” as its reverse), and an “incomplete collapse of independent insertions (iCII)” (and its reverse, an “incomplete creation of spurious independent insertions (iCSII)”) (see Fig. 5, panels e-h, for schematic illustrations on position-shift maps, and Additional file 1: Figure S6 on MSAs; see section *SM-5* of *Supplementary methods* in Additional file 1 for detailed definitions).^4^ We wrote a prototype Perl script that attempts to disentangle MSA errors in each erroneous segment by associating each position-shift block with a MSA error. The underlying idea is to compare indel events inferred by the two MSAs, and to attribute the changes in the inferred events to the moves of the blocks. (The simplest cases are illustrated in Fig. 5. See section *SM-6* of *Supplementary methods* in Additional file 1 for details. The Perl script is available as a part of Additional file 2.) In addition, we noticed some cases where a pair of errors (e.g., the “merge + iCII” in Additional file 1: Figure S1) is associated intrinsically with a pair of position-shift blocks. Our prototype Perl script also attempts to identify such pairs of errors (see section *SM-7* of *Supplementary methods* for details). If the script failed to associate a block with an error of any definite type in this way, the block was associated with a “complex” error.

Before performing the full-scale analysis, we manually validated the prototype script using the first five MSAs simulated along the tree of 15 mammals, in order to see if the script indeed works as intended. We confirmed that the script correctly classified 95% or more of 357 block-associated and block-pair-associated non-complex errors, 180 via MAFFT and 177 via Prank. (See Additional file 3 for more details.)

Tables 4, 5 and Additional file 1: Table S4 show the proportion of erroneous segments explained solely by each of these types of errors. Among them (except “complex”), “shift” was always the largest category. For erroneous segments in this category, it will be relatively easy to reach the true MSAs from the reconstructed MSAs. Its proportion decreased as the sequence divergence increases, from 79–90% for 12 primates to 19–32% for 9 fast-evolving mammals. In contrast, the proportion of “complex” increased with the divergence, from 4–8% for 12 primates to 55–67% for 9 fast-evolving mammals. Basically, the “complex” errors mean that their classification is beyond the scope of the current prototype script (see, e.g., Additional file 1: Figures S2 and S3). Thus, their percentage is expected to decrease in the future, as we develop an algorithm to parse position-shift maps more thoroughly.^5^ The third conspicuous category was “mixture,” in which each segment contains two or more different types of non-complex errors. Its proportion was about 3–10%. “Paired” accounted for substantial fractions (40–70%) of “mixture” when the divergence is small or moderate.

**Table 4:** Frequencies of errors of different types in MSAs among 12 primates

Error type	MAFFT (E-INS-1)	(Percent)	MAFFT (E-INS-i)	(Percent)	Prank (Best-fit)	(Percent)
Shift	113,658	(79.00%)	110,890	(80.01%)	120,802	(89.78%)
Merge	3779	(2.63%)	3920	(2.83%)	1875	(1.39%)
Purge	2175	(1.51%)	2198	(1.59%)	742	(0.55%)
Split	6	(0.004%)	1	(0.001%)	383	(0.29%)
Ex-nihilo	0	(0%)	4	(0.003%)	34	(0.03%)
v-Merge ^a^	926	(0.64%)	765	(0.55%)	489	(0.36%)
v-Split ^b^	51	(0.04%)	28	(0.02%)	142	(0.11%)
CII ^c^	1146	(0.80%)	1116	(0.81%)	110	(0.08%)
iCII ^d^	2191	(1.52%)	2335	(1.69%)	40	(0.03%)
Others ^e^	943	(0.66%)	900	(0.65%)	728	(0.54%)
Mixture ^f^	7814	(5.43%)	7304	(5.27%)	3774	(2.80%)
(Paired) ^g^	(5375)	(3.74%)	(5047)	(3.64%)	(2738)	(2.04%)
Complex ^h^	11,183	(7.77%)	9127	(6.59%)	5428	(4.03%)
Total	143,872	(100%)	138,588	(100%)	134,547	(100%)

NOTE: Shown in each cell is the number or the percentage of erroneous segments via a specified aligner (column) belonging to a specified error type (row). In each of the top 10 rows, the specified error type alone can explain each erroneous segment

^a^Vertical-merge

^b^Vertical-split

^c^Creation of independent insertions

^d^Incomplete creation of independent insertions

^e^Each segment is explained solely by a *non-complex* error type other than the 9 types above

^f^Each segment is explained by a mixture of two or more *non-complex* error types

^g^Each segment is explained solely by *non-complex* errors associated intrinsically with a pair, or pairs, of blocks. This is included in the “Mixture” category

^h^Each segment includes at least one complex error

Tables 4, 5 and Additional file 1: Table S4 also imply some differences in nature between errors via MAFFT (especially iterative) and those via Prank. (The progressive option of MAFFT was generally like its iterative option, but also somewhat similar to Prank.) Aside from the aforementioned three categories, relatively frequent categories were “merge,” “purge,” “iCII,” “CII” and “vertical merge” for MAFFT, and “merge,” “purge,” “vertical merge,” “split” and maybe “vertical split” for Prank. This was somewhat expected. First, “merge,” “purge” and “vertical merge” tend to increase both complete-likelihood and aligner-specific scores, by decreasing the number of indels, and thus are expected to be common regardless of the aligner. Second, “iCII” and “CII” frequently occur via MAFFT but not via Prank, because Prank was in a sense designed to reduce these types of errors, by appropriately scoring closely neighboring independent indels. And third, the relatively larger frequencies of “split” and “vertical split” via Prank may be a side effect of the aforementioned design. (Or, rather, it may be expected for a single-optimum-search aligner with a scoring scheme fairly close to the complete-likelihood.) Inspection of the “paired” category (Additional file 1: Table S5) also corroborated these observations.

**Table 5:** Frequencies of errors of different types in MSAs among 15 mammals

Error type	MAFFT (E-INS-1)	(Percent)	MAFFT (E-INS-i)	(Percent)	Prank (Best-fit)	(Percent)
Shift	118,707	(38.59 %)	130,029	(38.31 %)	199,756	(55.21 %)
Merge	6344	(2.06 %)	7832	(2.31%)	7026	(1.94 %)
Purge	6730	(2.19 %)	7954	(2.34 %)	5247	(1.45 %)
Split	18	(0.006 %)	5	(0.001 %)	1830	(0.51 %)
Ex-nihilo	1	(0.000 %)	0	(0 %)	138	(0.04 %)
v-Merge ^a^	1476	(0.48 %)	1391	(0.41 %)	2156	(0.60 %)
v-Split ^b^	190	(0.06 %)	56	(0.02 %)	598	(0.17 %)
CII ^c^	3131	(1.02 %)	4992	(1.48 %)	309	(0.09 %)
iCII ^d^	5315	(1.73%)	9150	(2.70 %)	141	(0.04 %)
Others ^e^	1311	(0.43 %)	1527	(0.45 %)	2225	(0.62 %)
Mixture ^f^	29,385	(9.55 %)	34,288	(10.10 %)	34,151	(9.44 %)
(Paired) ^g^	(11,813)	(3.84 %)	(13,193)	(3.89 %)	(14,718)	(4.07 %)
Complex ^h^	135,026	(43.89 %)	142,219	(41.90 %)	108,237	(29.92 %)
Total	307,634	(100 %)	339,446	(100 %)	361,814	(100 %)

The same note and footnotes apply as those for Table 4

**Fig. 6:**
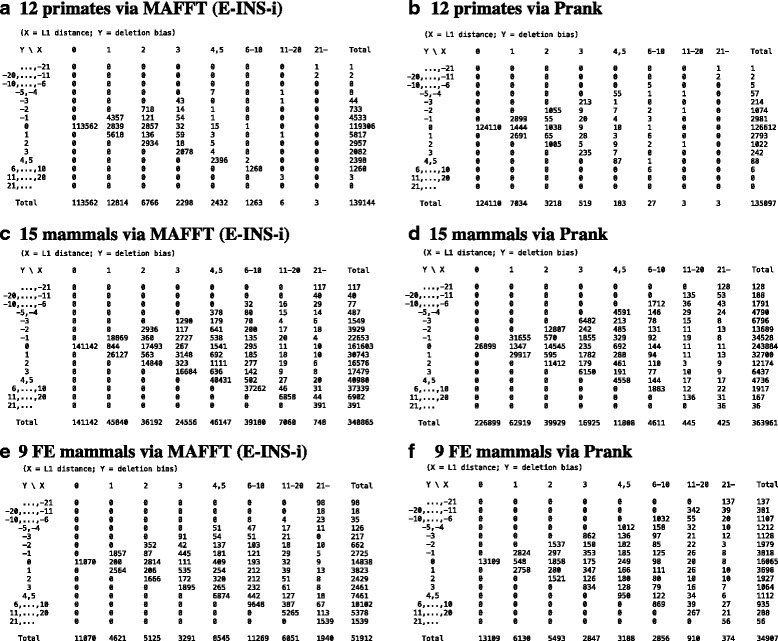
Different features of indel count misestimations by MAFFT (iterative) and Prank. **a,c,e** Via MAFFT, E-INS-i (i.e., iterative). **b,d,f** Via Prank. **a,b** With 12 primates. **c,d** With 15 mammals. **e,f** With 9 fast-evolving (FE) mammals. Each *panel* shows a 2-dimensional distribution of two measures of indel count misestimations, namely, the L1 distance (abscissa) and the deletion bias (ordinate). See section *M8* of *Methods* for the definitions of the measures. Each of the integers is the count of erroneous segments whose L1 distance and deletion bias belong to the specified classes

But these uncovered differences between the aligners are just the tip of the iceberg, and more differences will be revealed as more “complex” errors are disentangled. To get some hints on such differences, we also examined three measures of the misestimated indel counts. First, the “ordinary difference” is the number of indel events in the reconstructed MSA minus that in the true MSA. Second, the “L1 distance” is the summation of the differences between the two MSAs in the numbers of insertions and deletions. And third, the “deletion bias” measures the tendency to overestimate the number of deletions and underestimate the number of insertions. They are defined clearly in section *M8* of *Methods*. The 2-dimensional distributions of the L1 distance and the deletion bias (Fig. 6) highlight the different natures of errors via the two aligners. (The distributions for MAFFT (progressive) were quite similar to those for MAFFT (iterative) (Additional file 1: Figure S7).) Aside from “shifts” predominating the large population at the origin, (0, 0), errors via MAFFT (iterative) seem heavily biased toward overestimated deletions and/or underestimated insertions (panels A, C and E), whereas errors via Prank seem more balanced (panels B, D and F). This is evident also from the overall averages of the three measures (Additional file 1: Tables S6 and S7) and is consistent with the past findings [20, 28]. The result suggests that the tendencies directly revealed by the position-shift map analysis (Tables 4, 5 and Additional file 1: Table S4), i.e., the prevalence of CII-like and iCII-like errors via MAFFT and the prevalence of split-like and vertical-split-like errors via Prank, are likely to remain valid even after unraveling errors currently classified as “complex.”

The averages of the three measures behave differently also among the score categories (Additional file 1: Tables S6 and S7). For example, for MAFFT, the absolute value of the deletion bias is almost equal to the L1 distance in categories “I” and “D,” whereas the former is much smaller than the latter in category “S.” This result may imply the following composition of errors via MAFFT: categories “I” and “D” are relatively richer in CII-like and iCII-like errors, whereas category “S” is relatively richer in merge-like, purge-like and vertical-merge-like errors. Indeed, the score-category-wise distributions of error types showed a trace of such composition (data not shown).

### R6. Suggested strategies to improve accuracy of MSA reconstruction

The results of this study suggest that, even if we adhere to the traditional practice of searching for an optimum MSA, we could still substantially improve the accuracy of MSAs via the current state-of-the-art aligners. We could potentially correct about 15–75% of the MSA errors at the maximum. In the case of similarity-based aligners like MAFFT, especially after iterative refinement, the accuracy improvement would be only marginal by introducing a better MSA search strategy alone. Thus, we need to introduce the complete-likelihood score, or its good proxy, in order to cover most of the theoretically correctable MSA errors. In contrast, in the case of evolution-based aligners like Prank, the improvement by a better scoring system alone would be relatively small, and it is essential to explore the MSA space more thoroughly in order to substantially improve the accuracy. Because the current version of Prank performs solely the progressive alignment, introducing an iterative refinement step may significantly improve its accuracy.

Our analysis of position-shift maps showed that, among those true MSAs which were truly most likely but missed by the aligners, at least a majority could be identified by a simple exploration of the neighborhoods of the reconstructed MSAs, as long as the sequence divergences are at most moderate. On the other hand, the analysis also demonstrated that a substantial fraction of such true MSAs are quite “far away” from the reconstructed MSAs, especially in category “D” erroneous segments. To recover such true MSAs, we would need to devise a new algorithm that explores the MSA space to search preferentially for regions likely to harbor the true MSA. For this purpose, it would be helpful to delve further into the nature and characteristics of MSA errors, e.g., by using the position-shift map introduced in this study.

Another major conclusion of this study is that a substantial fraction (at least 25–85%) of MSA errors occur because the molecular evolutionary processes are stochastic by nature, that is, because the true MSA is not most likely (and thus not highest-scoring). This supports the hypothesis by Wong et al. [26] in a demonstrable manner. It also implies that, in order to recover most of the true MSAs, it is essential to obtain the probabilistic distribution of considerably likely MSAs, instead of just searching for a single most likely (or highest-scoring) MSA. This reemphasizes what have been concluded repeatedly in the past, especially concerning pairwise alignment methods (e.g., [54, 66]). Some algorithms aiming for this goal on MSAs were developed in the past (e.g., [55], and programs of this type are recently getting more and more practical (e.g., [28, 56–59]). But they seem to have been avoided by most biological researchers, possibly because the methods are formidably sophisticated, or maybe because people are skeptical of the usefulness or necessity of such methods. This study clarified that the probability distribution of MSAs is crucial in order to recover most, if not all, of the true MSAs. Moreover, our analyses via position-shift maps revealed that erroneously reconstructed MSAs in category “S” tend to be “closer” to the true MSAs than those in categories “I” and “D.” This, in conjunction with the fact that the former accounts for a substantial fraction of MSA errors, implies that even exploring only close neighborhoods of the reconstructed MSAs could recover a majority of true MSAs. Hence, it could lead to the programs conceptually and algorithmically more accessible. If such a program is established, the algorithm to calculate the complete-likelihood will further improve the accuracy of the prediction of MSAs. In the meantime, programs and methods that assess the reliability of the portions of an MSA, such as MUMSA [67], HOT, COS [68, 69], GUIDANCE [70], PSAR [71] and TCS [72], will continue to be useful, as long as the analysis’s focus is not on indels.

In this study, we focused our attention on MSAs of neutrally evolving DNA sequences. And we believe that the results presented here can be extrapolated to MSAs of weakly selected DNA sequences, such as non-coding sequences (e.g., [51]). However, the results may not be directly extrapolated all the way to MSAs of sequences under strong functional constraint, such as those coding for proteins with solid 3D structures. At this point, there seems to be a long way to go for simulating MSAs or calculating the ab initio occurrence probabilities of MSAs under a *realistic* evolutionary model of these strongly constrained sequences, although some recent simulators (such as INDELible [64]) and our theoretical formulation [37, 73] could provide a good starting point. Most benchmark MSA sets were based on structural alignments, and similarity-based aligners were in general tuned to perform excellently on these sets. Therefore, as far as the functions and the 3D structures are concerned, similarity-based aligners may provide decent solutions for the MSAs of strongly constrained sequences. When performing evolutionary analyses, however, it should always be kept in mind that CIIs, iCIIs and sequence analogies (due to convergent evolution) must not be confused with sequence homologies (due to shared ancestries).

### R7. Scope of this study

To keep the matters in perspective, it would be important to correctly understand the scope of this study.

First, the current versions of the tools presented here depend critically on correctly reconstructed gapless columns, and this poses some limitations on the MSAs (or the set of species) that can be handled. In a sense, the MSAs with the 9 fast-evolving mammals (Fig. 2c) could be deemed as largely beyond the scope of the current tools, because nearly a half of the erroneous segments were too long to be handled (item 7 of Table 2). But true MSAs themselves seemed generally tractable even with the 9 fast-evolving mammals (e.g., item 3 in Table 2). Thus, the essential problem must be the failure to correctly reconstruct gapless columns, rather than the lack of them. It depends on the total branch length; on average, as it increases, gapless segments become shorter and gapped segments become longer, making it harder to correctly reconstruct gapless columns. (For theoretical estimations, see section SM-8 of Supplementary methods in Additional file 1. Under the current simulation setting with the 9 fast-evolving mammals, the expected length was about 4.0 for a gapless segment and about 11.9 for a gapped segment.) It also depends on the individual branch lengths, because an alignment across a longer branch will be more difficult to reconstruct correctly. Provided that the total branch length can be kept nearly unchanged, increasing the number of species to divide long branches will make the MSAs more tractable, because more gapless columns will be correctly reconstructed. At least theoretically, we can even make the presented tools not depend on gapless columns, by exploiting the “phylogenetic correctness condition” on the ancestral sequence states (e.g., [34, 74]). Such a methodological improvement will be useful particularly for a MSA of many sequences connected by many short branches (even if the total branch length is large).

Second, for the MSA reconstruction and the score calculation, we used the true or the “best-fit” parameters and the true phylogenetic trees as guide trees, and we did not thoroughly examine the effects of wrong parameters on the MSA errors. In general, the accuracy of reconstructed alignments was shown to be robust against considerable perturbations of the parameters (except guide trees) [28, 66]. The effects of erroneous guide trees were also examined a number of times (e.g., [27, 70, 75, 76]). We refer the readers interested in the subjects to these studies.

Last but not least, it should be remembered that this study is based on MSAs simulated under simpler conditions than those expected in real, biological situations. Although we incorporated some biological realism by using the indel length distribution based on the past empirical studies, our simulation only modeled neutral substitutions and insertions/deletions (under 100 bases long). In contrast, real DNA sequences should also undergo more complex evolutionary processes such as long indels (over 100 bases long), selection, inversions, duplications, copy number changes of microsatellites, transposon insertions, etc. (e.g., [77–79]). We chose to focus on this simple setting because most aligners only take account of substitutions and indels, and because we expected that focusing on this basic setting would highlight the essence of the problems underlying MSA errors. In this way, we were able to avoid getting confounded by other biological complexities. We hope that this study will provide a ground on which more sophisticated studies on MSA errors could be conducted in the future, taking account of further biologically realistic features. An extended theoretical framework of sequence evolution [73] might help address the issues of MSAs affected by genomic rearrangements, among major biological complexities.

## Conclusions

In this study, to meticulously characterize MSA errors, we introduced two new tools, the complete-likelihood score and the position-shift map. The complete-likelihood score enables us to compare MSAs of the same homologous sequences in terms of their occurrence probabilities under the stochastic model of a genuine sequence evolution simulator. The position-shift map clearly visualizes MSA errors and could help disentangle a composite error into elementary ones. Our analyses of the simulated MSAs and their reconstructed counterparts revealed that a substantial fraction of MSA errors are due to the inherently stochastic nature of the evolutionary processes and thus could not be rectified even if we thoroughly searched the MSA space for the truly most likely MSA. This re-emphasizes how important it is to obtain a probability distribution of fairly likely MSAs, instead of merely searching for a single optimum MSA. The analyses also implied that, out of the remaining errors, most by the similarity-based aligners may be corrected via the complete-likelihood score or its good proxy, and most by the evolution-based aligners may be rectified via more thorough MSA space exploration such as in iterative refinement. This suggests the possibility to considerably improve the accuracy of MSA reconstruction even if adhering to the search for a single optimum MSA.

## Methods

### M1. Simulated MSAs

We created simulated MSAs via Dawg (version 1.2-RELEASE) [52] as explained in the following. First, out of a 36-species phylogenetic tree [65], we extracted three sub-trees of mammalian species, namely, the trees of 12 primates, 15 mammals, and 9 fast-evolving mammals. They were intended to represent the phylogenetic relationships with small, moderate and large sequence divergences, respectively, that we could commonly encounter in DNA sequence analyses (Fig. 2; Table 1). The correspondence between the sequence IDs and the species names is given in Additional file 1: Table S1. Pollard et al. [65] determined the branch lengths of their tree by the expected numbers of substitutions per 4-fold degenerate site, which are commonly used as a proxy of neutral evolutionary distances. Thus, we used the lengths with no modification for our simulations. Each simulation started with a random DNA sequence that is 1000 bases long. We used a Zipf power-law distribution of the indel length (*l*), *f*(*l*) = *l*^− 1.6^ [∑_*k* = 1_^∞^*k*^− 1.6^]^− 1^, which is among the empirically well established (see, e.g., [54] and references therein). We set the cut-off indel length of 100 bases to make the downstream analyses finish within a reasonable amount of time. The total indel rate was set at 1/8 = 0.125 indels/substitution, according to a data analysis of mammalian DNA sequences [80]. And the insertion rate was set equal to the deletion rate, broadly according to another data analysis on mammals [28]. Other simulation parameters were kept at the default, including the Jukes-Cantor base substitution model [81]. The Dawg control files used for the simulations are available as a part of Additional file 2. Each control file records a phylogenetic tree and other evolutionary model parameters.

### M2. MSA reconstruction

After removing all gaps from the simulated MSAs, the MSAs were reconstructed using MAFFT (version 7.154) [13–15] and Prank (version 130410) [19, 20]. The phylogenetic trees used for the simulations were fed into both aligners as guide trees. For both aligners, we also set the transition/transversion ratio at 1 and (if applicable) all base frequencies at 0.25, because we used the Jukes-Cantor model of base substitutions for our simulations. We set the other parameters as follows.

For MAFFT, we used an accuracy-oriented option, E-INS-i [15]. This option directs MAFFT to iteratively refine a MSA using a score to measure its consistency with pairwise alignments [14].^6^ It uses a local pairwise alignment algorithm with a general affine gap penalty [82]. We used this option because some of our simulated MSAs contain fairly long gaps (nearly 100 bases long). We set the maximum number of iteration cycles at 100 instead of the default value of 1000, to save computational time. Regarding the remaining parameters, we used default values because fine-tuning the parameters for MAFFT would take too much efforts and computational time and because the alignment accuracy is known to change only slightly under parameter perturbations (e.g., [28, 66]).

For Prank, we used two parameter settings. One is the default setting (except aforementioned parameters), including the gap-opening rate of 0.025 and the gap extension probability of 0.75. The other is the “best-fit” set of parameters, where the parameters of their HMM for indels (inevitably with a geometric length distribution) were fitted via a least-square method to the indel model (with the aforementioned power-law length distribution) used in each Dawg simulation. In both cases, we used the “+F” option, which forces already inferred insertions to be always skipped when calculating the indel score, as recommended by the developers [20]. Other parameters were kept at the default.

### M3. Pre-processing MSAs

In principle, MSA aligners can only reconstruct the “homology structure” of a MSA, which describes the mutual homology relationships among the residues (or sites) in the homologous sequences (e.g., [83]). Therefore, we pre-processed each of the true and reconstructed MSAs so that MSAs with the same homology structure will be represented identically. Details are given in section SM-2 of Supplementary methods in Additional file 1.

### M4. Partitioning true and reconstructed MSAs into correct and erroneous segments

After the pre-processing, we partitioned a pair of true and reconstructed MSAs into correctly and erroneously reconstructed segments, or “correct” and “erroneous” segments for short, in basically the same manner as in [27]. See section *SM-3* of *Supplementary methods* in Additional file 1 for more details.

### M5. Calculation of complete likelihood score and aligner-specific scores

For each of the true and reconstructed MSAs in each erroneous segment, the complete likelihood score was calculated as the summation of the logarithm of the total occurrence probability of the MSA’s gap configuration (the indel component) and the logarithm of the total occurrence probability of the MSA’s residue configuration (the substitution component). First, the indel component was computed using our first-approximate algorithm [61]. Briefly, the algorithm proceeds in four steps: (i) chopping the input MSA into gapped and gapless segments; (ii) enumerating all alternative parsimonious indel histories that can result in each gapped segment; (iii) calculating each gapped segment’s contribution to the probability by summing the probabilities over all parsimonious indel histories under a continuous-time Markov model of sequence evolution via indels [37, 62]; and (iv) calculating the total probability of the input MSA via indels as the product of an overall factor and the contributions from all gapped segments. This algorithm is based on a sound theoretical ground [37], and our extensive validation analyses [61, 62] demonstrated that the algorithm calculates the probabilities quite accurately under moderate conditions.^7^ Second, the substitution component is nothing other than the so-called “log-likelihood” of the substitution model, and can be calculated exactly using the pruning algorithm (e.g., [1, 2, 62]). It was computed via PhyML (version 20120412) [63] under the Jukes-Cantor model. Both components were computed with the phylogenetic tree and other parameters used for the simulation, including the power-law indel length distribution. Section SM-1 of Supplementary methods in Additional file 1 gives a theoretical proof that the complete-likelihood score can be calculated as the summation of the substitution component and the indel component, as long as some conditions are fulfilled.^1^

For each of the true and reconstructed MSAs in each segment, the MAFFT-specific score for the iterative refinement was calculated according to [13, 14], and the Prank-specific score was calculated according to [19, 20]. (The former was also used for the analysis of errors via E-INS-1, because our main purpose was to examine the effect of the iterative refinement.) It should be noted that both MAFFT and Prank calculate different MSA scores at different steps of their iterative refinement and progressive alignment, respectively. At different steps, the aligners align different pairs of sub-MSAs, across different branches or internal nodes. It is therefore inevitable that each MSA exhibits a number of different aligner-specific scores, whereas it has only a single complete-likelihood score. In this study, for each MSA in each erroneous segment, we simply summed aligner-specific scores, over the branches for MAFFT and over the nodes for Prank, and compared the reconstructed and true MSAs in terms of this summation. In each comparison, two scores were regarded as “equal” if they differ by less than 10^− 5^.

### M6. Partitioning position-shift map into position-shift blocks

For each erroneous segment, we created a “position-shift map” by assigning a position-shift to each residue of each sequence in either of the true and reconstructed MSAs (Fig. 1, panel c). The “position-shift” of a residue was defined as its horizontal position in the reconstructed MSA minus that in the true MSA. Then, the position-shift map was partitioned into “position-shift blocks” (panel d). Each position-shift block (or “block” for short) is a set of residues with the same position-shift that are contiguous along the alignment and the phylogeny. See section *SM-4* of *Supplementary methods* in Additional file 1 for more details.

### M7. Classifying MSA error associated with position-shift block

We examined each position-shift block in the reconstructed MSA and its counterpart in the true MSA, as well as their surrounding indel events, to see how the move of the block influenced the prediction of indel events via Dollo parsimony [84] (see, e.g., Fig. 5). Based on this examination, we judged whether the block is associated with a MSA error or not. If so, we classified the associated MSA error into “shift,” “merge,” “purge,” “split,” “ex-nihilo” (up to here from [27]), and a number of MSA-specific types (defined in section *SM-5* of *Supplementary methods* in Additional file 1). See section *SM-6* of *Supplementary methods* and Fig. 5 for more details on how a block was associated with an error. Out of those blocks that could not be associated with definite errors in this way, we attempted to associate a pair of blocks with a pair of definite errors. See section *SM-7* of *Supplementary methods* for more details.

### M8. Estimating indel counts and measures of indel count misestimation

For each of the true and reconstructed MSAs in each erroneous segment, we estimated the counts of insertions and deletions by averaging the counts over all parsimonious indel histories that could result in the MSA. The average was calculated with the histories’ relative occurrence probabilities as weights, using our recently developed method [61]. Let *Ct*(*ins*)_*Tr*_ and *Ct*(*del*)_*Tr*_ be the counts of insertions and deletions, respectively, in the true MSA. And let *Ct*(*ins*)_*Rec*_ and *Ct*(*del*)_*Rec*_ be these counts in the reconstructed MSA. Then, our three measures of the indel count misestimation, namely, the ordinary difference, the L1 distance, and the deletion bias, were defined as follows:

$$ \begin{array}{c}\hfill \left\{ Ordinary\kern0.5em  difference\right\}\kern0.5em \equiv \kern0.5em \left[Ct{(del)}_{Rec}-\kern0.5em Ct{(del)}_{Tr}\right]\kern0.5em +\kern0.5em \left[Ct(ins){}_{Rec}-\kern0.5em Ct{(ins)}_{Tr}\right],\hfill \\ {}\hfill \left\{L1\kern1em  distance\right\}\kern1em \equiv \kern0.5em \left|Ct{(del)}_{Rec}-\kern0.5em Ct{(del)}_{Tr}\right|\kern0.5em +\kern0.5em \left|Ct{(ins)}_{Rec}-\kern0.5em Ct{(ins)}_{Tr}\right|,\hfill \\ {}\hfill \left\{ Deletion\kern1em  bias\right\}\kern1em \equiv \kern0.5em \left[Ct{(del)}_{Rec}-\kern0.5em Ct{(del)}_{Tr}\right]\kern0.5em -\kern0.5em \left[Ct{(ins)}_{Rec}-\kern0.5em Ct{(ins)}_{Tr}\right].\hfill \end{array} $$

Here, |*Q*| denotes the absolute value of the number *Q*. The three measures were calculated for each erroneous segment. Then, they were tallied and averaged over all segments, or over segments in each particular category.

### M9. Program implementation

The Perl modules and main Perl scripts used in this study are available as a package named “ComplLiMment” (for “Complete-Likelihood from Multiple sequence alignment”) (version 0.6.1), which is archived in Additional file 2. The latest version of the package will be available in the “lolipog” directory at the FTP repository of http://Bioinformatics.Org [85].

### Availability of supporting data

The data sets supporting the results of this article are either included within the article and its additional files or reproducible via tools in Additional file 2.

## Additional files

Additional file 1:
**A PDF file that consists of Supplementary methods (sections SM-1 through SM-8), Tables S1, S2, S3, S4, S5, S6 and S7, and Figures S1, S2, S3, S4, S5, S6 and S7. Each figure is accompanied by a legend.** (PDF 5664 kb) Additional file 2:
**The ZIP archive of a package that contains the original versions of Perl modules and scripts used for creating the simulated and reconstructed MSAs and for analyzing these MSAs. **It also contains a README file that describes how to use the modules and scripts. (The modules and scripts will run on a Mac OS X terminal. And it will probably run on other UNIX platforms, although we have not tested whether they indeed do.) The package also contains the Dawg control files that were used for the simulations. The latest version of the package (“ComplLiMment_P.verxxx”) can be found in the ‘lolipog’ directory at the FTP repository of the Bioinformatics Organization [85]. (ZIP 4746 kb) Additional file 3:
**A ZIP archive, containing the results of our manual validation analyses of our prototype Perl script to classify MSA errors into various types.** See “README.txt” contained in it for its synoptic explanation. (ZIP 1247 kb)

## Abbreviations

(i)CII: (incomplete) collapse of independent insertions
(i)CSII: (incomplete) creation of spurious independent insertions
DP: dynamic programming
HMM: hidden Markov model
indel: insertion/deletion
MSA: multiple sequence alignment
PWA: pairwise (sequence) alignment

## Footnotes

1 It has been known that, under some probabilistic models of indels that are not evolutionary models and in addition under equilibrium of any time-reversible substitution model, the logarithm of the total likelihood of a MSA can be calculated like this (see, e.g., [86]). (Regarding the independence of the likelihood of a substitution model from details on indel histories, the idea dates back at least to [1].) *SM-1* shows that the calculation can be performed also under genuine stochastic evolutionary models and even under non-equilibrium (and thus even with non-time-reversible substitution models), as long as the (possibly time-dependent) residue frequencies satisfy certain conditions.

2 This, however, does not necessarily mean that Prank’s scoring system approximates the true likelihood itself accurately. In fact, our recent analyses [60] showed that the standard hidden Markov model (HMM) of indels, which Prank’s scoring system is based on, substantially underestimates the true occurrence probabilities of alignments involving overlapping indels and/or indels longer than several residues. This problem could be serious when we try to identify truly most likely MSAs involving such indels (as indicated by our position-shift map analyses) or when we need an accurate probability distribution of MSAs.

3 In some papers (e.g., [66, 87]), a “shift,” a “merge,” and a “purge” are called a “gap wander,” a “gap attraction,” and a “gap annihilation,” respectively.

4 The CII, and possibly also iCII, were already discussed in previous studies (e.g., [19, 20, 28]), and the authors of these studies called them “collapsed insertions,” or simply “gap-attractions.” We coined the terms “CII” and “iCII” because we considered them to depict the phenomena better.

5 Theoretically, we could always fully disentangle any composite error (that involves at least one “complex” error) by considering a path of erroneous moves, each of which is defined by a single position-shift block, that leads from the true MSA to the reconstructed one. Actually, based on this idea, our prototype script attempts to associate a pair of definite errors to a pair of blocks (see section *SM-7* of *Supplementary methods*). However, if, e.g.*,* an erroneous segment contains 5 non-background blocks, we will have to consider 5! = 120 possible paths. Because it could take very long to consider all such paths, and because we were currently unsure of how we should deal with such a large number of alternative paths, this “full solution” was not implemented in this study.

6 For the purpose of calculating the consistency scores of MSA segments later, we slightly modified MAFFT so that it would output pairwise homology relationships it infers.

7 As confirmation, we also performed a brief validation analyses on the current three sets of simulated MSAs (Fig. 2; Table 1). Out of the gapped segments without long apparent indels (item 2 minus item 3 in Table 2), those with ancestral states consistent with no parsimonious indel history accounted for only 0.1%, 0.2% and 1.6% for 12 primates, 15 mammals and 9 fast-evolving mammals, respectively. And the true and predicted values of the relative frequencies among alternative parsimonious indel histories matched almost perfectly (with the correlation coefficients 0.9994, 0.99999 and 0.99999, respectively). The results reassured us that the algorithm would work well on the current three sets of MSAs.
