# Supplementary material for: Characterization of multiple sequence alignment errors using complete-likelihood score and position-shift map
Source: BMC Bioinformatics. 2016 Mar 18;17:133. doi: 10.1186/s12859-016-0945-5 (PMC4799563; doi:10.1186/s12859-016-0945-5)
Supplement: Additional file 1: — A PDF file that consists of Supplementary methods (sections SM-1 through SM-8), Tables S1, S2, S3, S4, S5, S6 and S7, and Figures S1, S2, S3, S4, S5, S6 and S7. Each figure is accompanied by a legend. (PDF 5664 kb) [file 12859_2016_945_MOESM1_ESM.pdf]

Additional file 1 of
"Characterization of multiple sequence alignment errors using complete-likelihood score and position-shift map,"

by Kiyoshi Ezawa

Table of contents

Supplementary methods **pp. 2-17**

SM-1. Complete-likelihood score: definition and "indel-substitution factorization" pp.2-12

SM-2. Pre-processing MSAs: details p.12

SM-3. Partitioning pair of MSAs into correct and erroneous segments: details pp.12-13

SM-4. Partitioning position-shift map into position-shift blocks: details pp.13-14

SM-5. MSA-specific error types pp.14-15

SM-6. Associating MSA error with single position-shift block pp.15-17

SM-7. Associating pair of MSA errors with pair of position-shift blocks pp.17-19

SM-8. Theoretical mean lengths of gapless and gapped segments pp.19-21

Additional references **p. 21**

Supplementary tables **pp. 22-29**

Supplementary figures (with legends) **pp. 30-39**

Supplementary methods

SM-1. Complete-likelihood score: definition and "indel-substitution factorization"

This section provides one of the key results in this study, that is, the formal proof that the complete-likelihood score can be calculated as the summation of the indel component and the substitution component, under genuine stochastic evolutionary models. Briefly, the proof proceeds as follows: (I) Express the occurrence probability of a given MSA as the summation of probabilities over all possible evolutionary processes that can result in the MSA; (II) divide the set of all evolutionary processes into subsets, each of which consists of processes sharing the same indel process; (III) show that the total probability of each subset over all consistent substitution processes is independent of the shared indel process, as long as it is evolutionarily consistent with the MSA; (IV) by factoring out the indel-independent total probability over substitution processes, express the probability of the MSA as the product of the total probability over substitution processes (under a fixed indel process) and the total probability over indel processes; and (V) take the logarithm of the total probability. In the following, we give details on the proof. As you will see shortly, part (III) is the main part of the proof, and it is rather long. So, it was divided into 10 paragraphs, (III-1) through (III-10). [NOTE: In this section, as in our recent study [73], $\breve{s} \equiv (s, \vec{\omega})$ denotes an "extended" sequence state, which consists of a "basic" state (s) that changes only through insertions/deletions (indels), and the residue configuration ($\vec{\omega}$) that fills in the basic state. (See below for more details.)]

(Preparation 1) Consider a given MSA, $\alpha[\breve{s}_1, \breve{s}_2, ..., \breve{s}_k]$, of k (DNA or protein) sequences, $\breve{s}_1, \breve{s}_2, ..., \breve{s}_k$. (Let $\breve{\alpha}$ be the abbreviation of $\alpha[\breve{s}_1, \breve{s}_2, ..., \breve{s}_k]$.) Consider also that the phylogenetic tree, $T = \left(\{n\}\big|_T, \{b\}\big|_T\right)$, of the aligned sequences is also given. (Here n and b represent a node and a branch (an edge), respectively. And n^R denotes the root node hereafter.) Then, consider a given genuine stochastic evolutionary model, $\Theta = (\Theta_S, \Theta_{ID})$. Here Θ_S and Θ_{ID} denote an evolutionary model of substitutions and that of indels, respectively, equipped with specific parameters. The occurrence probability, $P\left[\breve{\alpha} \,\middle|\, \Theta, T\right]$, of MSA $\breve{\alpha}$ conditioned on this model setting, Θ, (and the tree, T,) is interpreted as the "complete-likelihood," $L\left(\Theta, T\right)\big|_{\breve{\alpha}}$, that is, the likelihood of the given "complete" evolutionary model, Θ (, and the given tree, T), given the MSA, $\breve{\alpha}$:

$$L\left(\Theta, T\right)\big|_{\breve{\alpha}} \equiv P\left[\breve{\alpha} \,\middle|\, \Theta, T\right]. \qquad \text{--- Eq.(1)}$$

In this paper, we deal with a general continuous-time Markov model as the specific genuine

stochastic evolutionary model, Θ.

(Preparation 2) Previously (in Appendix A3 of [73]), we proved that, if some conditions are satisfied, the above "complete-likelihood" is factorable into the product of the "basic" component and the "residue" component. Here the "basic" component is the total probability of the possible indel processes, and the "residue" component is the total probability of the possible substitution processes (including the initial residue states). Thus, we will call this factorization the "indel-substitution factorization." The proof given there was considerably long, because of two major reasons. (1) It started with a quite general model and gradually narrowed down its scope, in order to find the conditions for the factorization. And (2) it was described in terms of detailed expressions on the general continuous-time Markov model, including the exponential of the time-integration of its rate operator. Here, we will give a substantially shorter proof of the indel-substitution factorization, by (1') imposing some simplifying conditions from the beginning, and by (2') minimizing the use of expressions on the general continuous-time Markov model. Throughout this section, however, we will tacitly assume that we are working with a general continuous-time Markov model (satisfying some conditions). In the model, a sequence state, $\breve{s}$, is represented as $\breve{s} = (s, \vec{\omega})$. Here s is the basic state, which is an array of sites in the sequence. (Each site may be assigned an ancestry and possibly other attributes.) And $\vec{\omega} = (\omega_1, \omega_2, ..., \omega_{L(s)}) \in \Omega^{L(s)}$ is a vector of residues that fill in the sites of the sequence. Here $L(s)$ is the number of sites in s and Ω is the set of residue types. (See Appendix A1 of [73].) As in [37,60,61,73], a "history" is an untimed record of a series of fixed mutation events. Here, however, we mainly consider evolutionary processes, each of which is a timed record of the series of fixed mutation events. When we refer to a "summation" over processes, we tacitly assume that it includes the multiple-time integration over the timings of the events as well. (For brevity, an evolutionary process is represented by adding a dot over the symbol of its evolutionary history counterpart. Similarly, a set of evolutionary processes is represented by adding a dot over the symbol of the corresponding set of evolutionary histories.)

(I) Generalizing the proof given in sections 3.1 & 3.2 of [37], we can show that the complete-likelihood can be calculated by summing the occurrence probabilities (more precisely, probability densities) of all evolutionary processes that potentially give rise to the MSA, $\breve{\alpha}$:

$$L(\Theta, T)\big|_{\breve{\alpha}} = \sum_{\dot{\psi} \in \dot{\Psi}[\breve{\alpha}; T]} P\left[\dot{\psi} \mid \Theta, T\right] \quad . \qquad \text{--- Eq.(2)}$$

Here, $\dot{\Psi}\left[\breve{\alpha}; T\right]$ is the set of all evolutionary processes (including the root sequence state

$\breve{s}^R = (s^R, \vec{\omega}^R)$) along tree T that can result in MSA $\breve{\alpha}$. And each evolutionary process, denoted as $\dot{\breve{\psi}}$ here, is made up of four components: $\dot{\breve{\psi}} = \left(s^R, \vec{\omega}^R, \dot{\psi}_{ID}, \dot{\psi}_S\right)$. Here, s^R is the basic state of the root sequence, $\vec{\omega}^R$ is its residue state, $\dot{\psi}_{ID}$ is an indel process, and $\dot{\psi}_S$ is a substitution process. These four components are not completely independent of each other. First, because of the causality, $\dot{\psi}_{ID}$ and $\dot{\psi}_S$ depend on $\breve{s}^R$, but not *vice versa*. Second, the substitution process depends on the indel process, because an indel process determines the presence/absence of the sites at which substitution events take place. Explicitly taking account of these dependences, the above evolutionary process could be represented as: $\dot{\breve{\psi}} = \left(s^R, \vec{\omega}^R(s^R), \dot{\psi}_{ID}(s^R), \dot{\psi}_S(s^R, \vec{\omega}^R, \dot{\psi}_{ID})\right)$. We also notice that the above set, $\dot{\breve{\Psi}}\left[\breve{\alpha}; T\right]$, can be expressed as:

$$\dot{\breve{\Psi}}\left[\breve{\alpha}; T\right] = \left\{ \dot{\breve{\psi}} = \left(s^R, \vec{\omega}^R, \dot{\psi}_{ID}, \dot{\psi}_S\right) \middle| \begin{array}{l} s^R \in S\left[\alpha; n^R; T\right], \quad \vec{\omega}^R \in \Omega^{L(s^R)}, \\ \dot{\psi}_{ID} \in \dot{\mathrm{H}}^{ID}\left[\alpha; s^R; T\right], \\ \dot{\psi}_S \in \dot{\breve{\mathrm{H}}}^S\left[\breve{\alpha}; \dot{\psi}_{ID}; \breve{s}^R = (s^R, \vec{\omega}^R); T\right] \end{array} \right\} . \quad \text{--- Eq.(3)}$$

Here, α $(= \alpha\left[s_1, s_2, ..., s_k\right])$ is the "skeleton," *i.e.*, the "basic" component, of MSA $\breve{\alpha} = \alpha\left[\breve{s}_1, \breve{s}_2, ..., \breve{s}_k\right]$. $S\left[\alpha; n^R; T\right]$ is the set of all "basic" sequence states at the root (n^R) that are consistent with the tree (T) and α. $\dot{\mathrm{H}}^{ID}\left[\alpha; s^R; T\right]$ is the set of all indel processes along T that start with s^R at the root and that can give rise to α. And $\dot{\breve{\mathrm{H}}}^S\left[\breve{\alpha}; \dot{\psi}_{ID}; \breve{s}^R = (s^R, \vec{\omega}^R); T\right]$ is the set of all substitution processes along T that start with $\breve{s}^R = (s^R, \vec{\omega}^R)$ at the root, that are consistent with $\dot{\psi}_{ID}$, and that can "flesh out" α to give rise to $\breve{\alpha}$.

(II) To go further, we first formally decompose Eq.(3) as follows:

$$\dot{\breve{\Psi}}\left[\breve{\alpha}; T\right] = \bigcup_{\substack{\dot{\breve{\psi}}_{ID} = (s^R, \dot{\psi}_{ID}) \\ \in \dot{\breve{\Psi}}_{ID}[\alpha; T]}} \left\{ (\dot{\breve{\psi}}_{ID}, \dot{\breve{\psi}}_S) \middle| \dot{\breve{\psi}}_S = (\vec{\omega}^R, \dot{\psi}_S) \in \dot{\breve{\Psi}}_S\left[\breve{\alpha}; \dot{\breve{\psi}}_{ID}; T\right] \right\} . \quad \text{--- Eq.(4a)}$$

Here,

$$\dot{\breve{\Psi}}_{ID}\left[\alpha; T\right] \equiv \left\{ \dot{\breve{\psi}}_{ID} = (s^R, \dot{\psi}_{ID}) \middle| s^R \in S\left[\alpha; n^R; T\right],\ \dot{\psi}_{ID} \in \dot{\mathrm{H}}^{ID}\left[\alpha; s^R; T\right] \right\} \quad \text{--- Eq.(4b)}$$

is the set of all pairs, each of a "basic" state at the root and an indel process along T, that can give rise to α. And

$$\dot{\breve{\Psi}}_S\left[\breve{\alpha};\dot{\breve{\psi}}_{ID};T\right] \equiv \left\{\dot{\breve{\psi}}_S=(\vec{\omega}^R,\dot{\psi}_S)\;\middle|\; \begin{array}{l}\vec{\omega}^R\in\Omega^{L(s^R)},\\ \dot{\psi}_S\in\dot{\breve{\mathrm{H}}}^S\left[\breve{\alpha};\dot{\psi}_{ID};\vec{s}^R=(s^R,\vec{\omega}^R);T\right]\end{array}\right\} \quad \text{--- Eq.(4c)}$$

is the set of all pairs, this time each of a "residue" state at the root and a substitution process along T, that are compatible with $\dot{\breve{\psi}}_{ID}$ and that can give rise to $\breve{\alpha}$ by filling out α with the extant residues. Second, let us recall a basic identity in the probability theory:

$$P\left[A,B\,|\,C\right]\equiv\frac{P\left[A,B,C\right]}{P\left[C\right]}=\frac{P\left[B,C\right]\times P\left[A\,|B,C\right]}{P\left[C\right]}=P\left[B\,|\,C\right]\times P\left[A\,|B,C\right]\;. \quad \text{--- Eq.(5)}$$

By using this, we decompose the summand on the right hand side of Eq.(2) as follows:

$$P\left[\dot{\breve{\psi}}=(\dot{\breve{\psi}}_{ID},\dot{\breve{\psi}}_S)\,|\,\Theta,T\right]=P\left[\dot{\breve{\psi}}_{ID}\,|\,\Theta,T\right]\times P\left[\dot{\breve{\psi}}_S\,\middle|\,\dot{\breve{\psi}}_{ID},\Theta,T\right]. \quad \text{--- Eq.(6)}$$

Now, substituting Eq.(4a) and Eq.(6) into Eq.(2), we get:

$$L(\Theta,T)\big|_{\breve{\alpha}} = \sum_{\dot{\breve{\psi}}_{ID}\in\dot{\breve{\Psi}}_{ID}[\alpha;T]}\left\{P\left[\dot{\breve{\psi}}_{ID}\,|\,\Theta,T\right]\times\sum_{\dot{\breve{\psi}}_S\in\dot{\breve{\Psi}}_S\left[\breve{\alpha};\dot{\breve{\psi}}_{ID};T\right]}P\left[\dot{\breve{\psi}}_S\,\middle|\,\dot{\breve{\psi}}_{ID},\Theta,T\right]\right\}. \quad \text{--- Eq.(7)}$$

This is the general equation that holds true regardless of details of the evolutionary model ($\Theta=(\Theta_S,\Theta_{ID})$), and it provides the sound starting point of the following argument.

(III-1) To make Eq.(7) easier to handle, we need some assumptions. (See section 1 of [73].) First, we assume the following condition.

Condition (i): "The indel rates are independent of the residue state and the substitution process before each indel event."

Under this condition, the probability (density) of an indel process becomes independent of the substitution model setting, that is:

$$P\left[\dot{\breve{\psi}}_{ID}\,|\,\Theta=(\Theta_S,\Theta_{ID}),T\right]=P\left[\dot{\breve{\psi}}_{ID}\,|\,\Theta_{ID},T\right]. \quad \text{--- Eq.(8)}$$

Next, we assume the following two conditions.

Condition (ii): "The substitution rates at each site are independent of the states (both "basic" and residue) and the evolutionary processes (both indel and substitution) at other sites."

Condition (iii): "The probability of the residue state of an inserted subsequence, conditioned on the insertion, can be factorized into the product of residue probabilities over the inserted sites. And the probabilities are independent of sequence states before insertion."

In an equation, the condition (iii) could be expressed as:

$$p_I\left(\delta\vec{\omega}'=(\omega'_{x+1},\ldots,\omega'_{x+l});x,l;s,t_I\right)=\prod\nolimits_{i=1}^{l}p_I\left(\omega'_{x+i};\upsilon_{x+i}(s'),t_I\right). \quad \text{--- Eq.(9)}$$

Here $p_I\left(\delta\vec{\omega}'=(\omega'_{x+1},...,\omega'_{x+l}); x,l; s, t_I\right)$ denotes the probability, conditioned on the insertion of l sites between the x th and the $x+1$ th sites of s at time t_I, that the inserted residue state is $\delta\vec{\omega}'=(\omega'_{x+1},...,\omega'_{x+l})$. And $p_I\left(\omega'_{x+i}; \upsilon_{x+i}(s'), t_I\right)$ denotes the probability, conditioned on the insertion of the site with ancestry $\upsilon_{x+i}(s')$ (, which is the $x+i$ th site of the new sequence s',) at t_I, that the inserted residue is ω'_{x+i}. Under the conditions (ii) and (iii), we can perform the following factorization:

$$\sum_{\dot{\psi}_S \in \dot{\Psi}_S\left[\breve{\alpha};\dot{\psi}_{ID};T\right]} P\left[\dot{\psi}_S \,\middle|\, \dot{\psi}_{ID}, \Theta, T\right] = \prod_{\upsilon \in \Upsilon\left[\breve{\alpha};T\right]} \left\{ \sum_{\dot{\psi}_S[\upsilon] \in \dot{\Psi}_S\left[\breve{\alpha}[\upsilon];\dot{\psi}_{ID}[\upsilon];T\right]} P\left[\dot{\psi}_S[\upsilon] \,\middle|\, \dot{\psi}_{ID}[\upsilon], \upsilon, \Theta, T\right] \right\} .$$

--- Eq.(10)

Here, $\Upsilon\left[\breve{\alpha}, T\right]$ is the set of ancestries (across T) assigned to the columns of $\breve{\alpha}$. $\dot{\psi}_S[\upsilon]$ is a substitution process (including the initial residue state) at the site with ancestry υ, and $\dot{\psi}_{ID}[\upsilon]$ is the component of $\dot{\psi}_{ID}$ involving the site with υ. $\breve{\alpha}[\upsilon]$ is the MSA column that corresponds to the site with υ. And $\dot{\Psi}_S\left[\breve{\alpha}[\upsilon]; \dot{\psi}_{ID}[\upsilon]; T\right]$ is the set of all substitution processes (including the initial residue states) along T that are compatible with $\dot{\psi}_{ID}[\upsilon]$ and that can give rise to $\breve{\alpha}[\upsilon]$ by filling out the "skeleton" ($\alpha[\upsilon]$) with the extant residues. (Eq.(10) could be derived similarly to the factorization for indel processes described in section 4 of [37].)

(III-2) Now, consider the site-wise probability on the right hand side of Eq.(10), *i.e.*,

$$P\left[\breve{\alpha}[\upsilon] \,\middle|\, \dot{\psi}_{ID}[\upsilon], \upsilon, \Theta, T\right] \equiv \sum_{\dot{\psi}_S[\upsilon] \in \dot{\Psi}_S\left[\breve{\alpha}[\upsilon];\dot{\psi}_{ID}[\upsilon];T\right]} P\left[\dot{\psi}_S[\upsilon] \,\middle|\, \dot{\psi}_{ID}[\upsilon], \upsilon, \Theta, T\right]. \quad \text{--- Eq.(11)}$$

The conditions (ii) and (iii) guarantee that, under a fixed indel process component involving each site, the substitution processes at the site can be described by a continuous-time Markov model. Thus, the probability could be calculated similarly to the column-wise probability under a standard continuous-time Markov model of substitutions (e.g., [1,2,62]). Here, we generalize the argument to non-equilibrium situations and time-dependent models. [NOTE: If we consider that the inserted residue probabilities belong to the substitution model (Θ_S), the probability of a substitution process ($\dot{\psi}_S[\upsilon]$) could depend on the indel model (Θ_{ID}) only through the indel process $\dot{\psi}_{ID}[\upsilon]$. Hence we could replace Θ in Eq.(11) with Θ_S.]

(III-3) Eq.(11) could be expressed differently depending on whether the site existed at the root (n^R) or not. (For details, see Appendix A3 of [73].) If the site existed at the root,

we have:

$$P\left[\breve{\alpha}[\upsilon]\,\middle|\,\dot{\breve{\psi}}_{ID}[\upsilon],\upsilon,\Theta_S,T\right]$$

$$= \sum_{\omega^R\in\Omega} P\left[(\omega^R,n^R)\,\middle|\,\upsilon,\Theta_S\right] \times \prod_{b\in \mathrm{B}_P^{IN}[\dot{\breve{\psi}}_{ID}[\upsilon],T]} \left\{ \sum_{\omega^D(b)\in\Omega} P\left[(\omega^D(b),n^D(b))\,\middle|\,(\omega^A(b),n^A(b))\right] \right\}$$

$$\times \prod_{b'\in \mathrm{B}_D[\dot{\breve{\psi}}_{ID}[\upsilon],T]} \left\{ \sum_{\omega_D(b')\in\Omega} P\left[(\omega_D(b'),t_D(b'))\,\middle|\,(\omega^A(b'),n^A(b'))\right] \right\}$$

$$\times \prod_{b''\in \mathrm{B}_P^{X}[\dot{\breve{\psi}}_{ID}[\upsilon],T]} P\left[(\omega^D(b''),n^D(b''))\,\middle|\,(\omega^A(b''),n^A(b''))\right] \;.$$

--- Eq.(12a)

Here and in Eq.(12b) below, we should consider that the summation over residue states at each node actually involves all the probabilities whose conditions or outcomes concern the states at the node. (We used this notation because there was no neater way to precisely represent the multiple summations here.) In Eq.(12a), $\mathrm{B}_P^{IN}\left[\dot{\breve{\psi}}_{ID}[\upsilon],T\right]$ is the set of branches in the tree (T) along which the site (with ancestry υ) persists under the indel process $\dot{\breve{\psi}}_{ID}[\upsilon]$ and whose lower-ends are internal nodes. $\mathrm{B}_P^{X}\left[\dot{\breve{\psi}}_{ID}[\upsilon],T\right]$ is the set of site-persisting branches whose lower-ends are external nodes (with extant sequences). And $\mathrm{B}_D\left[\dot{\breve{\psi}}_{ID}[\upsilon],T\right]$ is the set of branches along which the site is deleted. $P\left[(\omega^R,n^R)\,\middle|\,\upsilon,\Theta_S\right]$ is the probability that the residue state is ω^R at n^R, given Θ_S and υ. $P\left[(\omega^D(b),n^D(b))\,\middle|\,(\omega^A(b),n^A(b))\right]$ is the probability that the residue is $\omega^D(b)$ at the lower-end (*i.e.*, descendant node) $n^D(b)$ of branch b, given that the residue is $\omega^A(b)$ at the upper-end (*i.e.*, ancestral node) $n^A(b)$ of the branch. Similarly, $P\left[(\omega_D(b'),t_D(b'))\,\middle|\,(\omega^A(b'),n^A(b'))\right]$ is the probability that the residue is $\omega_D(b')$ at time $t_D(b')$ when the site is deleted along branch b', again given that the residue is $\omega^A(b')$ at $n^A(b')$. We symbolically omitted but tacitly assumed the dependence of these conditional probabilities on Θ_S and υ. It should also be kept in mind that each node has a single residue state under each substitution process, as long as the subject site existed at the node.

(III-4) If the site did not exist at the root but instead was inserted at time $t_I(b_I)$ along branch b_I, we have (see Eq.(A3.12c) of [73]):

$$
\begin{aligned}
&P\left[\breve{\alpha}[\upsilon] \,\middle|\, \dot{\breve{\psi}}_{ID}[\upsilon], \upsilon, \Theta_S, T\right] \\
&= \sum_{\omega_I \in \Omega} p_I\left(\omega_I; \upsilon, t_I(b_I)\right) \times \sum_{\omega^D(b_I) \in \Omega} P\left[(\omega^D(b_I), n^D(b_I)) \,\middle|\, (\omega_I, t_I(b_I))\right] \\
&\quad \times \prod_{b \in \mathrm{B}_P^{IN}[\dot{\breve{\psi}}_{ID}[\upsilon], T]} \left\{ \sum_{\omega^D(b) \in \Omega} P\left[(\omega^D(b), n^D(b)) \,\middle|\, (\omega^A(b), n^A(b))\right] \right\} \\
&\quad \times \prod_{b' \in \mathrm{B}_D[\dot{\breve{\psi}}_{ID}[\upsilon], T]} \left\{ \sum_{\omega_D \in \Omega} P\left[(\omega_D, t_D(b')) \,\middle|\, (\omega^A(b'), n^A(b'))\right] \right\} \\
&\quad \times \prod_{b'' \in \mathrm{B}_P^{X}[\dot{\breve{\psi}}_{ID}[\upsilon], T]} P\left[(\omega^D(b''), n^D(b'')) \,\middle|\, (\omega^A(b''), n^A(b''))\right] \quad .
\end{aligned}
$$

--- Eq.(12b)

Here, as in Eq.(9), $p_I\left(\omega_I; \upsilon, t_I(b_I)\right)$ is the probability that the site (with ancestry υ) has residue ω_I when inserted, given that it was inserted at $t_I(b_I)$ along b_I. And $P\left[(\omega^D(b_I), n^D(b_I)) \,\middle|\, (\omega_I, t_I(b_I))\right]$ is the probability that the residue is $\omega^D(b_I)$ at the lower-end $n^D(b_I)$ of b_I, given that the residue was ω_I when the site was inserted along b_I. Again, we tacitly assumed the dependence of the conditional probabilities on Θ_S and υ.

(III-5) Now, we will show that, under some additional conditions, Eqs(12a,b) are identical, and independent of the details on the indel processes ($\dot{\breve{\psi}}_{ID}[\upsilon]$'s), as long as the processes are consistent with a given MSA column, $\breve{\alpha}[\upsilon]$ (or, more precisely, a given "skeleton," $\alpha[\upsilon]$). For this purpose, we recall the "phylogenetic correctness" condition (*e.g.*, [34,74]), which has to be satisfied by any indel processes (or their resulting ancestral "basic" states) that could result in a given MSA column. In the present context, the "phylogenetic correctness" condition could be rephrased as follows.

"When a site exists at two points on the tree (T), it must also exist all along the path on T that connects these points."

Given $\alpha[\upsilon]$, the simplest indel history satisfying this condition could be found via the Dollo parsimony principle [84], which searches for the history with the fewest indels while restricting the number of insertions to at most one but allowing an unlimited number of deletions. Given $\alpha[\upsilon]$, we can easily reconstruct the Dollo parsimonious history (denoted as $\breve{\psi}_{ID}^0\left[\alpha[\upsilon]\right]$ hereafter) by making the site exist along all paths connecting the external nodes holding the site, and by making the site absent from all remaining parts of the tree. The other indel histories (and processes) that satisfy the "phylogenetic correctness" condition could be constructed by continuously extending one or more paths of "site existence" from the point(s) on the "web" of paths of $\breve{\psi}_{ID}^0\left[\alpha[\upsilon]\right]$, while keeping the site existence/absence at the external

nodes intact.

(III-6) Thus, all we have to prove is that we can retract such extended paths back to the Dollo parsimonious history while retaining the site-wise probability (Eq.(12a) or Eq.(12b)) unchanged. Broadly speaking, there are two types of extended paths: one upward, extending toward the root (n^R); and the other downward, extending toward (but short of) external nodes where the site is absent. We first retract the downward paths, and then we retract the upward one.

(III-7) Each downward extension ends either at a point along a branch (like $t_D(b')$) or at an internal node (like $n^D(b)$). In any case, the end of each downward extension is associated with a summation of the single conditional probabilities, like: $\sum_{\omega' \in \Omega} P\left[(\omega', \tau) \,\middle|\, (\omega^A(b), n^A(b))\right]$, where $\tau = t_D(b)$ or $\tau = n^D(b)$. By the definition of the conditional probabilities, this summation is always 1 (unity). Therefore, we can indeed retract each downward extension until it becomes a point (node). The indel process resulting from all such retractions is the Dollo parsimonious process, maybe bearing an upward extension.

(III-8) The upward extension also ends either at a point along a branch (like $t_I(b_I)$) or at an ancestral node (like $n^A(b)$, especially n^R). We need to separately consider the cases where the site was already present at the root (Eq.(12a)) and those where the site was inserted (Eq.(12b)). In the latter case, the extension could be retracted without any effect on the probability if the following condition is satisfied:

Condition (iv): "For the inserted residue probabilities at the site with ancestry υ, $\left\{p_I(\omega; \upsilon, \tau)\right\}_{\omega\in\Omega,\, \tau\in T}$, we have:

$$\sum_{\omega' \in \Omega} \left\{p_I(\omega'; \upsilon, \tau') \times P\left[(\omega, \tau) \,\middle|\, (\omega', \tau')\right]\right\} = p_I(\omega; \upsilon, \tau), \qquad \text{--- Eq.(13)}$$

for all upward paths on T (, whose upper-end and lower-end are denoted as τ' and τ, respectively)."

This is because the summation over residues at an internal node (n') on an un-branched path ($[\tau', \tau]$) could be trivially performed as:

$$\sum_{\omega' \in \Omega} P\left[(\omega', n') \,\middle|\, (\omega_1, \tau')\right] \times P\left[(\omega_2, \tau) \,\middle|\, (\omega', n')\right] = P\left[(\omega_2, \tau) \,\middle|\, (\omega_1, \tau')\right], \quad \text{--- Eq.(14)}$$

as a result of the Chapman-Kolmogorov equation. When the upward extension reaches the root (n^R), we could retract the extension if the following equation is satisfied:

$$\sum_{\omega' \in \Omega} \left\{ P\left[(\omega', n^R) \mid \upsilon, \Theta_S\right] \times P\left[(\omega, n^D(b_I^0)) \,\middle|\, (\omega', n^R)\right] \right\} = p_I\left(\omega; \upsilon, n^D(b_I^0)\right). \quad \text{--- Eq.(15)}$$

Here b_I^0 is the branch where the site is inserted in the Dollo parsimonious history. Comparing Eq.(15) to Eq.(13) with the substitutions $\tau' = n^R$ and $\tau = n^D(b_I^0)$, we see that, along with Eq.(13), the following equation is necessary and sufficient.

Condition (v): $P\left[(\omega, n^R) \mid \upsilon, \Theta_S\right] = p_I\left(\omega; \upsilon, n^R\right)$. --- Eq.(15')

The conditions (iv) and (v) could be rephrased in the following words.

"The inserted residue probabilities at the root, $\left\{p_I\left(\omega; \upsilon, n^R\right)\right\}_{\omega \in \Omega}$, are identical to the initial residue probabilities, $\left\{P\left[(\omega, n^R) \mid \upsilon, \Theta_S\right]\right\}_{\omega \in \Omega}$. And the inserted residue probabilities along the tree, $\left\{p_I\left(\omega; \upsilon, \tau\right)\right\}_{\omega \in \Omega,\, \tau \in T}$, also evolve according to the continuous-time Markov model of substitutions at the site (with ancestry υ)."

These conditions were automatically satisfied in most of the past evolutionary models with indels (*e.g.*, [88-91]), because these models used time-reversible substitution models and assumed that the inserted residue probabilities are given by the equilibrium frequencies of the substitution model.

(III-9) Some indel histories leave "null" MSA columns consisting only of gaps. Usually, they do not appear in an input MSA. Let us consider Eq.(12a) or Eq.(12b) compatible with an indel history resulting in such a null column. After retracting all the downward extensions in such a case, we are left with a point, which is either the root (n^R) or an insertion point ($t_I(b_I)$). In the former case the site-wise probability reduces to $\sum_{\omega^R \in \Omega} P\left[(\omega^R, n^R) \mid \upsilon, \Theta_S\right]$, and in the latter case it reduces to $\sum_{\omega_I \in \Omega} p_I\left(\omega_I; \upsilon, t_I(b_I)\right)$. Either way, the probability becomes 1 (unity). Thus, the "null" MSA columns give trivial probabilities due to substitutions.

(III-10) Thus, we proved that, under the conditions (iv) and (v) (*i.e.*, under Eq.(13) and Eq.(15')), the site-wise probabilities, Eq.(12a) and Eq.(12b), do not depend on the details on $\dot{\psi}_{ID}[\upsilon]$. Thus, under these conditions, $P\left[\breve{\alpha}[\upsilon] \,\middle|\, \dot{\psi}_{ID}[\upsilon], \upsilon, \Theta, T\right]$ in Eq.(11) becomes equal to $P\left[\breve{\alpha}[\upsilon] \,\middle|\, \breve{\psi}_{ID}^0\left[\alpha[\upsilon]\right], \upsilon, \Theta, T\right]$, where $\breve{\psi}_{ID}^0\left[\alpha[\upsilon]\right]$ is the Dollo parsimonious indel history for the column $\alpha[\upsilon]$. Substituting this result into Eq.(10), we have:

$$\sum_{\dot{\psi}_S \in \dot{\Psi}_S[\breve{\alpha};\dot{\psi}_{ID};T]} P\left[\dot{\psi}_S \,\middle|\, \dot{\psi}_{ID}, \Theta, T\right] = \prod_{\upsilon \in \Upsilon[\breve{\alpha};T]} P\left[\breve{\alpha}[\upsilon] \,\middle|\, \breve{\psi}^0_{ID}[\alpha[\upsilon]], \upsilon, \Theta_S, T\right] . \quad \text{--- Eq.(10')}$$

The right hand side of Eq.(10') depends only on $\breve{\alpha}$ but not on other details of $\dot{\psi}_{ID}$, because the set of Dollo parsimonious histories, $\left\{\breve{\psi}^0_{ID}[\alpha[\upsilon]]\right\}_{\upsilon \in \Upsilon[\breve{\alpha};T]}$, is uniquely determined by α.

Recalling the conditions (ii) and (iii), we could re-interpret the right-hand side of Eq.(10') as the probability of $\breve{\alpha}$, given α, Θ_S and T. This also conforms to the standard calculation of the probability of a MSA via substitutions (*e.g.*, [1,2,62]). Thus, we have:

$$\sum_{\dot{\psi}_S \in \dot{\Psi}_S[\breve{\alpha};\dot{\psi}_{ID};T]} P\left[\dot{\psi}_S \,\middle|\, \dot{\psi}_{ID}, \Theta, T\right] = P\left[\breve{\alpha} \,|\, \alpha, \Theta_S, T\right] = L(\Theta_S, T)\big|_{\breve{\alpha}} . \quad \text{--- Eq.(10'')}$$

On the right hand side, we omitted α from the argument of the likelihood, because α trivially follows from $\breve{\alpha}$.

(IV) Substituting Eq.(8) and Eq.(10'') into Eq.(7), the complete-likelihood can be rewritten as:

$$\begin{aligned} L(\Theta, T)\big|_{\breve{\alpha}} &= \sum_{\dot{\psi}_{ID} \in \dot{\Psi}_{ID}[\alpha;T]} \left\{ P\left[\dot{\psi}_{ID} \,|\, \Theta_{ID}, T\right] \times L(\Theta_S, T)\big|_{\breve{\alpha}} \right\} \\ &= \left\{ \sum_{\dot{\psi}_{ID} \in \dot{\Psi}_{ID}[\alpha;T]} P\left[\dot{\psi}_{ID} \,|\, \Theta_{ID}, T\right] \right\} \times L(\Theta_S, T)\big|_{\breve{\alpha}} . \end{aligned} \quad \text{--- Eq.(7')}$$

Because $\dot{\Psi}_{ID}[\alpha;T]$ is the set of all indel processes (including the "basic" root states) that can give rise to α, the summation on the right hand side of Eq.(7') is actually the probability of α under given Θ_{ID} and T:

$$\sum_{\dot{\psi}_{ID} \in \dot{\Psi}_{ID}[\alpha;T]} P\left[\dot{\psi}_{ID} \,|\, \Theta_{ID}, T\right] = P\left[\alpha \,|\Theta_{ID}, T\right] = L(\Theta_{ID}, T)\big|_{\alpha} . \quad \text{--- Eq.(16)}$$

Substituting Eq.(16) into Eq.(7'), we obtain the final result:

$$L(\Theta, T)\big|_{\breve{\alpha}} = L(\Theta_{ID}, T)\big|_{\alpha} \times L(\Theta_S, T)\big|_{\breve{\alpha}} . \quad \text{--- Eq.(7'')}$$

Thus, *provided that the conditions (i)-(v) are satisfied*, the "complete likelihood" of the entire evolutionary model ($\Theta = (\Theta_S, \Theta_{ID})$) (and a tree T) under a given MSA ($\breve{\alpha}$) can be factorized into the product of two likelihoods. One is the likelihood of the substitution model (Θ_S), which can be exactly calculated via the widely accepted pruning algorithm (*e.g.*, [1,2,62,63]). And the other is the likelihood of the indel model (Θ_{ID}), which can be calculated quite accurately via our *ab initio* perturbative formulation of the general continuous-time Markov model [37,60,61].

(V) Taking the logarithms of both sides of Eq.(7''), we see that the logarithm of the

complete likelihood (called the "complete-likelihood score" here) is the summation of the logarithms of the two likelihoods.

SM-2. Pre-processing MSAs: details

In principle, MSA aligners can only reconstruct the "homology structure" of a MSA, which describes the mutual homology relationships among the residues (or sites) in the homologous sequences (*e.g.*, [83]). They cannot predict further details concerning the indel processes that produced the sequences. Therefore, we pre-processed each of the true and reconstructed MSAs so that MSAs with the same homology structure will be represented identically. Specifically, we first removed MSA columns consisting only of gaps. Then, we swapped two adjacent blocks, each consisting only of the columns with the same gap pattern, when they satisfied the following two conditions. (1) A block contains residues only in sequences that contain exclusively gaps in the other block. (In other words, the set of sequences with residues in one block does not overlap that in the other.) (2) Viewed in the MSA, the highest sequence with a residue in the left block is higher than that in the right block. The swapping was re-iterated until no adjacent pair of blocks was found swappable.

SM-3. Partitioning pair of MSAs into correct and erroneous segments: details

We partitioned a pair of true and reconstructed MSAs into correctly and erroneously reconstructed segments (or "correct" and "erroneous" segments for short) in a manner similar in philosophy to but slightly different from that by [27], by a column-by-column comparison of the two MSAs. It should be noted, however, that there are a few differences between the current method and the method in [27]. (i) In this study, we also distinguished gaps between different pairs of residues in each sequence. (ii) When an erroneously reconstructed segment begins or ends in the middle of a gapped segment, it was extended to accommodate the entire gapped segment. This guarantees correct calculation of the complete likelihood score. And (iii) if the two MSAs give an identical set of inferred indel histories, the segment was reclassified as "correct."

Here we detail how we partitioned a MSA pair. First, in each of the true and reconstructed MSAs, each residue of each sequence was assigned a number, which is the count of residues on its left along the sequence. Then, each gap character ("-") was also assigned a number, which is the average of the numbers assigned to the residues on its immediate left and right. (For example, we assign 10.5 to all gaps in a run of gaps sandwiched by the residues numbered 10 and 11.) This happens to be effectively equivalent to the "pos" recoding of gap characters [92], and to the "evol" recoding (*ibid.*) when dealing with

position-shift blocks (see sections *M6* and *M7* of *Methods* in the main text and *sections SM-3 and SM-5* below). This way, each column of each MSA is equipped with a vector of the assigned numbers, which are arranged in the same order as the sequences in the MSA. The true and reconstructed MSAs were then compared column by column. Two columns were judged as "equivalent" if both give the same vector. According to this column-wise comparison, the pair of MSAs was tentatively partitioned into an alternating series of correct and erroneous segments. Each correct segment is a contiguous set of pairs of equivalent columns. Each erroneous segment is a pair of segments, one of which is a contiguous set of columns in the true MSA whose equivalents were not found, and the other is its counterpart in the reconstructed MSA. Then, if a tentative erroneous segment ends in the middle of a gapped segment (*i.e.*, a segment of MSA consisting of contiguous gapped columns), the erroneous segment was extended to encompass the entire gapped segment. Accordingly, the neighboring correct segment was retracted. This process is necessary for the correct calculation of MSA scores, especially the complete-likelihood scores. Then, finally, we examined whether or not the pair of MSAs in each tentative erroneous segment were actually equivalent, potentially resulting from an identical set of local indel histories. Such equivalence typically involves different representations of two or more independent insertions (or a deletion followed by an insertion) that occurred between two successive correct segments (see, *e.g.*, Figure 5 of [37]). When the pair turned out to be equivalent, the tentative "erroneous" segment was re-classified as "correct," and it was merged with the flanking "correct" segments.

SM-4. Partitioning position-shift map into position-shift blocks: details

As can be seen from panel C of Figures 1, and S1-S3, a position-shift map usually has a clear block structure, where each block with a position-shift is delimited by two positions along the MSA and one or more branches in the tree. There could be a number of strategies to identify the blocks. In this study, we employed a "bottom-up" strategy, in which small blocks were constructed first, and then they were merged to form larger blocks. An important thing is how to handle some common exceptions, such as gaps in a block, multiple independent yet exactly aligned blocks sharing a position-shift, nested blocks, and a block interrupted by insertions along remote branches. The detailed procedure is as follows.

Given a position-shift map, we first chopped each sequence into segments, each of which consists of contiguous residues with the same position-shift. When two segments with the same position-shift were separated by a run of gaps alone, they were merged. Second, looking across the sequences, the segments with the same position-shift and sharing the same

start and end coordinates (in the MSA) were merged into a tentative block, and its boundaries on the phylogenetic tree were examined. If the set of sequences were delimited by the boundaries fewer than the sequences, the tentative block was established. Otherwise, the sequence set was split into two sub-sets at their most recent common ancestor, and the boundaries were examined again. This "split-and-examine" process continued until the boundaries become fewer than the sequences in every sub-set. Third, if three blocks with the same position-shift enclosed another block with a different position-shift, the three blocks were merged into a larger block. And, finally, if two or more neighboring blocks with different position-shifts were separated only by insertions along non-delimiting branches, and if the blocks had consistent position-shifts, they were merged into a "composite block" (like the blue and red blocks in Figure S2 D).

SM-5. MSA-specific types of errors

In addition to the error types in pairwise alignments (PWAs), namely, "shift," "merge," "split," "purge," and "ex-nihilo" [27] (see panels A-D of Figure 5), we newly defined the following "elementary" error types (requiring only a single block-wise move) that can only occur in MSAs (panels E-H of Figure 5). The definitions were based on the actual (anecdotal) comparisons between true and reconstructed MSAs and on the brief considerations of their likeliness, based on the resulting changes in the MSA scores. The following definitions are just the translations into words of the intuitive definitions (in Figure S6) and their equivalents (when the tree gets unrooted).

(1) A "vertical merge," which has two types. In one type, two neighboring deletion-derived gaps along two sibling branches are erroneously aligned together to form a wrong gap derived from a spurious deletion along the parent branch (Figure S6 A,B). In the other type, a correct gap was derived from an insertion along a branch, and another correct gap was derived from a neighboring deletion along one of the branch's children. Then, they are erroneously aligned together to form a wrong gap derived from a spurious insertion along the other child of the branch.

(2) A "vertical split," which is the reverse of a vertical merge. In other words, a "vertical split" becomes a "vertical merge" if the true and reconstructed MSAs swap their roles.

(3) A "collapse of independent insertions (CII)," which has two types. In one type, two neighboring equally long inserted sequences along two branches (neither siblings nor parent-child) are erroneously aligned together to form multiple

wrong gaps derived from multiple spurious deletions along other branches (Figure S6 C,D). In the other type, two equally long sequences, one aligned with a gap derived from a deletion along a branch, and the other created by a neighboring insertion along one of the branch's grandchild or more remote descendant, are erroneously aligned together. In a modified version of CII, instead of the relevant sequences themselves, their flanking sequences, or one relevant sequence and the flanking sequence of the other, are erroneously aligned together.

(4) A "creation of spurious independent insertions (CSII)," which is the reverse of a CII.

(5) An "incomplete collapse of independent insertions (iCII)," which is similar to CII, but occurs between gaps of different lengths (Figure S6 E,F), whereas a CII occurs between equally long gaps.

(6) An "incomplete creation of spurious independent insertions (iCSII)," which is the reverse of an iCII.

(7) We also defined some other types, such as "partial merge/split," "partial vertical merge/split," "overshoot-merge/split," etc. See the subroutine, "classify_error_assoc_w_sgl_cblk," of the prototype Perl script, "classify_msa_errors_via_mblks.alpha2.pl," in the "ComplLiMment" package (in Additional file 2) for detailed conditions to define them.

Errors that do not fall into these categories were tentatively classified as "complex." (See *SM-7* for more details on the "complex" errors.)

SM-6. Associating MSA error with single position-shift block

The idea underlying the procedures in this and the next sections is to compare two indel histories, one inferred from the true MSA and the other from the reconstructed MSA, and to attribute the history differences to the moves of position-shift blocks (see, *e.g.*, Figure 5). In general, the move of a block will change the predictions on indels along its delimiting branches and/or their neighboring branches. Thus, the key is to identify such indels from the inferred histories. The following is the detailed procedure.

Specifically, we used a pair of position-shift maps, one on the true MSA and the other on the reconstructed MSA, and examined the move of each position-shift block between the two maps and the resulting changes in the inferred indel history. For simplicity, we here used the Dollo parsimonious indel histories [84] inferred from the two MSAs. For each

position-shift block, we also attempted to determine a "main" branch that delimits the block. If the block had only one delimiting branch, it became the one. If the block had two or more delimiting branches, we first excluded those that were already the "main" delimiting branches of other blocks. If only one branch remained, it was used for the "main" delimiting branch; otherwise, we suspended the examination of the block. Then, for each block in each MSA, we listed "involved" indel events, each of which is defined as an event satisfying two conditions: (1) it either overlaps or flanks the block (along the MSA); and (2) it occurred either along the main delimiting branch or along its parent, child, or sibling. An "involved" indel event in a MSA was removed from the list if it had its equivalent in the other MSA. Finally, we judged what type of MSA error is associated with the block by comparing the two lists of indel events "involved" with the block, one in the true MSA and the other in the reconstructed MSA. In the following, we will explain some specific example cases.

(i) The error was regarded as a "shift" if the block in each MSA was flanked by only one event along the main delimiting branch, and if the corresponding events in the two MSAs were equally long, of the same type (*i.e.*, insertion or deletion), and on opposite sides of the block (Figure 5 A).

(ii) It was regarded as a "merge" in either of the following two cases. (1) If the blocks in the true and reconstructed MSAs involved two events and one event, respectively, all of the same type, and if the length of the latter event is the summation of the lengths of the former events (Figure 5 B). (2) If the block in the true MSA involved two events of opposite types, if the block in the reconstructed MSA involved one event that is of the same type as the longer event in the true MSA, and if the length of the reconstructed event equals the difference of the lengths of the true events (Figure 5 C).

(iii) It was regarded as a "split" in an "opposite" case from either of (ii), that is, if we observed nearly the same situation as either of the cases in (ii), with the only difference that the roles of true and reconstructed MSAs were swapped.

(iv) It was regarded as a "purge" if the block in the true MSA involved two events, of opposite types and of an equal length, and if the block in the reconstructed MSA involved no events (Figure 5 D).

(v) It was regarded as an "ex-nihilo" in an opposite case from (iv).

(vi) It was regarded as a "vertical merge" if the block in the true MSA involved two equally long events, one along the delimiting branch and the other along its parent, child, or sibling branch, and if the block in the reconstructed MSA involved only a single event along yet another branch which is also the parent,

child, or sibling of the delimiting branch (Figure 5 E, F).

(vii) It was regarded as a "vertical split" in an opposite case from (vi).

(viii) It was regarded as a "collapse of independent insertions (CII)" if the following three conditions are satisfied. (a) The block in the true MSA was flanked from one end by an insertion along a branch that is not the delimiting branch, its parent, child, or sibling. (b) The block was also overlapped by another equally long insertion on the other end and along the delimiting branch. And (c) both insertions disappeared and instead multiple spurious deletions emerged on the former end of the corresponding block in the reconstructed MSA (Figure 5 G). Similarly, a CII was called also in a case where the true MSA resulted from a deletion along a branch and an insertion along its grandchild or more remote descendant (see item (3) in *SM-5*).

(ix) It was regarded as a "creation of spurious independent insertions (CSII)" in an opposite case from (viii).

(x) It was regarded as an "incomplete collapse of independent insertions (iCII)" in a case similar to (viii), with the only difference that the two inserted sequences in the true MSA were not equally long (and therefore only one insertion disappeared in the reconstructed MSA) (Figure 5 H).

(xi) It was regarded as an "incomplete creation of spurious independent insertions (iCSII)" in an opposite case from (x).

We actually defined more cases in which an error associated with a block was considered to be of a definite type. [The precise conditions for the error-type vs. block associations are implemented in the subroutine, "classify_error_assoc_w_sgl_cblk," of the prototype Perl script, "classify_msa_errors_via_mblks.alpha2.pl," in the "ComplLiMment" package (in Additional file 2).]

SM-7. Associating pair of MSA errors with pair of position-shift blocks

After the first round of analysis described in *SM-6*, we re-examined those position-shift blocks that could not be associated with any errors of definite types. Here we tentatively refer to such a block as a "complex block." And we refer to a block whose examination was suspended in the first round as a "suspended block." Broadly speaking, after identifying pairs of seemingly interacting blocks, we first considered two series (or "paths") of moves of two blocks in each pair, second "restored" an intermediate MSA in each series by undoing the move of the second block in the series, third compared the intermediate MSA with both the

true and the reconstructed MSAs, and finally chose the series explained by a simpler pair of errors. The following is the detailed procedure.

First, we noticed some cases in each of which a complex or suspended block (like the purple block in Figure 1, panel D) could be absorbed into a neighboring block (like the blue one) to form a larger block. Thus, we absorbed a complex or suspended block (called "block A" here) into another block (called "block B" here), which is either complex or associated with a definite error, if the following four conditions were fulfilled. :

(a) The two blocks have the same shift.

(b) The clades of the sequences involved with the two blocks are phylogenetically neighboring each other.

(c) Block A is completely aligned with block B, that is, there should be no part of block A horizontally sticking out of block B in either reference or reconstructed MSA.

(d) The set of indel events involved with block A is completely included in the event set involved with block B, regarding both MSAs.

After excluding the absorbed blocks, we attempted to form pairs of blocks out of the remaining complex blocks and blocks associated with definite errors. For this purpose, we first made a graph by linking two blocks that involve the same indel event. Then, we formed clusters, each of which consists of all blocks that are directly or indirectly linked with one another. Then, we picked only clusters of two blocks each as "block-pairs." (From the following analysis, we excluded those pairs that consist only of blocks associated with definite errors.)

Finally, we attempted to associate each block-pair with a pair of errors of definite types (as in Figure S1) in the following manner. (Here, the components of the subject block-pair will be referred to as "block C" and "block D.") For the block-pair, we obtained two "intermediate MSAs" from the reconstructed MSA: one (denoted as "rec-C") by undoing the move of block C, and the other (denoted as "rec-D") by undoing the move of block D. The two intermediate MSAs correspond to two different paths of block-wise moves, both starting from the reference MSA and leading to the reconstructed MSA. For example, "rec-C" could occur in a path where block D moves before the move of block C. Thus, we attempted to associate block D with a definite error by comparing the indel events inferred from the reference MSA with those from "rec-C," and to associate block C with a second definite error by comparing the events inferred from "rec-C" with those from the reconstructed MSA. Each association was attempted just as in *SM-6*. If successful, this associates the block-pair with a pair of definite errors. Also, if successful, the use of "rec-D" associates the block-pair with another pair of definite errors. If both attempts were successful, the simpler pair of errors was

chosen. If only one attempt was successful, the resulting pair of errors was associated to the block-pair. If neither attempt was successful, the results of the first round were retained.

After these analyses were completed, some complex blocks could neither be associated with definite errors nor be incorporated into any other block. We regarded these blocks as involved in “complex” errors.

SM-8. Theoretical mean lengths of gapless and gapped segments

The current versions of both methods, one to calculate the complete likelihood score and the other to characterize MSA errors via position-shift maps, depend on the existence of correctly reconstructed gapless columns, though they are not absolute requisites of the methods themselves. In addition, in order to avoid extremely long computation of the gap component of the complete-likelihood scores, we set an upper bound (say, L_{U1} bases) on the length of a gapped segment. For a meaningful characterization of erroneous segments, it is also necessary that the erroneous segments only rarely contain gapped segment longer than L_{U1}. Here let us calculate the mean lengths of a gapless segment and a gapped segment.

For simplicity, we will consider a space-homogeneous indel model of Dawg [52]. (For more general cases, see [37].) Let λ_I and λ_D be the total rates of insertions and deletions, respectively, per site per expected substitution. Then, the total rate that a sequence of length L experiences an insertion or a deletion is: $R_X(L) = \lambda_I(L-1) + \lambda_D(L-1+\bar{l}_D)$, where $\bar{l}_D$ is the mean deletion length. [NOTE: This “exit rate” is smaller than that in [52] by $2\lambda_I$, because we consider insertions at either end of the subject sequence as irrelevant.] Thus, the probability that a sequence of length L has experienced no indel throughout a phylogenetic tree (T, with total branch length $|T|$) is:

$$P_{no-indel}(L;T) = \exp\{-|T|\, R_X(L)\} = \exp\left\{-|T|\left(\lambda_I(L-1) + \lambda_D(L-1+\bar{l}_D)\right)\right\}. \quad \text{--- Eq.(SM-8.1)}$$

From this, the probability that a particular ancestral site results in a gapless column is:

$$P_{GLC} \equiv P\left[gapless\ column \mid T\right] = P_{no-indel}(L=1;T) = \exp\left(-|T|\,\lambda_D\,\bar{l}_D\right). \quad \text{--- Eq.(SM-8.2)}$$

And the probability that a column flanking a gapless column is also gapless is:

$$P_{FGLC} \equiv P\left[flanking\ gapless\ column \mid gapless\ column, T\right] = \exp\left(-|T|\,(\lambda_D + \lambda_I)\right).$$

--- Eq.(SM-8.3)

Using Eqs.(SM-8.2,3), we have:

$$P_{no-indel}(L;T) = P_{GLC}\,\left(P_{FGLC}\right)^{L-1} \quad \text{--- Eq.(SM-8.1')}$$

From Eqs.(SM-8.1,3), the probability to have a gapless segment of length L at a particular position is:

$$P_{GLS}(L\,;T) = (1-P_{FGLC})^2\, P_{no-indel}(L\,;T) = \left(1-P_{FGLC}\right)^2 P_{GLC}\left(P_{FGLC}\right)^{L-1}. \quad \text{--- Eq.(SM-8.4)}$$

Along an ancestral sequence of length L_A, there are ($L_A - L + 1$) positions where a gapless segment of length L can reside. When L_A is sufficiently long, the variable part, $-L+1$, becomes negligible. Thus, the mean length of a gapless segment is approximately given by:

$$\bar{L}_{GLS}(T) \approx \left[\sum_{l=1}^{L_A} l\, P_{GLS}(l;T)\right] \Big/ \left[\sum_{l=1}^{L_A} P_{GLS}(l;T)\right] \approx 1 \Big/ \left(1-P_{FGLC}\right) \text{. --- Eq.(SM-8.5)}$$

Among the L_A sites of the ancestral sequence, $L_A\, P_{GLC}$ sites are expected to give gapless columns. Thus, on average, there should be $\bar{N}_{GLS} = L_A\, P_{GLC} / \bar{L}_{GLS}(T) \approx L_A\, P_{GLC}\left(1-P_{FGLC}\right)$ gapless segments along the ancestral sequence. Using these quantities, we can roughly calculate the average number of ancestor-derived columns in a *gapped* segment as:

$$\begin{aligned}\bar{N}_{ADC\in GS}(T) &\approx L_A(1-P_{GLC}) / \bar{N}_{GLS} \approx (1-P_{GLC}) \big/ \left[P_{GLC}\left(1-P_{FGLC}\right)\right] \\ &= \left[\exp\left(|T|\,\lambda_D\,\bar{l}_D\right)-1\right] \big/ \left[1-\exp\left(-|T|\,(\lambda_D+\lambda_I)\right)\right] \quad .\end{aligned} \quad \text{--- Eq.(SM-8.6)}$$

In general, however, gapped segments contain insertion-derived columns as well. At present, we don’t know exactly how many such columns should be. Nevertheless, by resorting to a *time-reversed* counterpart of the evolutionary model, the number may be approximated as follows:

$$\bar{N}_{IDC\in GS}(T) \approx \left[\exp\left(|T|\,\lambda_I\,\bar{l}_I\right)-1\right] \big/ \left[1-\exp\left(-|T|\,(\lambda_D+\lambda_I)\right)\right] \quad . \quad \text{--- Eq.(SM-8.7)}$$

Here $\bar{l}_I$ denotes the mean insertion length. Combining Eqs.(SM-8.6,7), we get a rough estimation of the average size of a *gapped* segment:

$$\begin{aligned}&\bar{L}_{GS}(T) = \bar{N}_{ADC\in GS}(T) + \bar{N}_{IDC\in GS}(T) \\ &\approx \left[\exp\left(|T|\,\lambda_D\,\bar{l}_D\right) + \exp\left(|T|\,\lambda_I\,\bar{l}_I\right) - 2\right] \big/ \left[1-\exp\left(-|T|\,(\lambda_D+\lambda_I)\right)\right] \quad .\end{aligned} \quad \text{--- Eq.(SM-8.8)}$$

In the limit $|T| \mapsto 0$, Eq.(SM-8.8) reduces to $\bar{L}_{GS}(T) \mapsto \left(\lambda_D\,\bar{l}_D + \lambda_I\,\bar{l}_I\right) \big/ \left(\lambda_D + \lambda_I\right)$, as expected. Moreover, in the limit $|T| \mapsto \infty$, $\bar{L}_{GS}(T)$ approaches infinity, also as expected.

Additional references

88. Thorne JL, Kishino H, Felsenstein J. An evolutionary model for maximum likelihood alignment of DNA sequences. J Mol Evol. 1991;33:114-124.

89. Thorne JL, Kishino H, Felsenstein J. Inching toward reality: an improved likelihood model of sequence evolution. J Mol Evol. 1992;34:3-16.

90. Miklós I, Lunter GA, Holmes I. A “long indel” model for evolutionary sequence alignment. Mol Biol Evol. 2004;21:529-540.

91. Rivas E, Eddy SR. Probabilistic phylogenetic inference with insertions and deletions. PLoS Comput Biol. 2008;4:e1000172.

92. Blackburne BP, Whelan S. Measuring the distance between multiple sequence alignments. Bioinformatics 2012;28:495-502.

Supplementary tables

Table S1. Correspondence between sequence IDs and species names

12 primates		15 mammals		9 fast-evolving mammals	
Sequence ID	Species name	Sequence ID	Species name	Sequence ID	Species name
prim001	human	mamm001	human	fema001	human
prim002	chimpanzee	mamm002	chimpanzee	fema002	galago
prim003	orangutan	mamm003	colobus monkey	fema003	mouse
prim004	gibbon	mamm004	baboon	fema004	rabbit
prim005	colobus monkey	mamm005	macaque	fema005	cow
prim006	vervet	mamm006	dusky titi	fema006	hdgehog
prim007	baboon	mamm007	owl monkey	fema007	shrew
prim008	macaque	mamm008	marmoset	fema008	tenrec
prim009	dusky titi	mamm009	mouse lemur	fema009	rock hyrax
prim010	owl monkey	mamm010	galago		
prim011	marmoset	mamm011	cow		
prim012	squirrel monkey	mamm012	dog		
		mamm013	horseshoe bat		
		mamm014	armadillo		
		mamm015	elephant		

NOTE: They were extracted from the 36-species tree of [65].

Table S2. Frequencies of three broad score categories

Aligner	MAFFT (progressive) [a]	MAFFT (iterative) [a]	Prank
12 primates			
I	19967 (13.8%)	6320 (4.5%)	17238 (12.8%)
D	28937 (20.0%)	32719 (23.5%)	2864 (2.1%)
S	95510 (66.1%)	100096 (71.9%)	114994 (85.1%)
Overall	144414 (100%)	139135 (100%)	135096 (100%)
15 mammals			
I	103934 (33.7%)	43296 (12.7%)	123919 (34.1%)
D	106917 (34.6%)	172786 (50.7%)	26407 (7.3%)
S	97928 (31.7%)	124674 (36.6%)	213546 (58.7%)
Overall	308779 (100%)	340756 (100%)	363872 (100%)
9 fast-evolving mammals			
I	13513 (56.5%)	5486 (10.6%)	15530 (44.7%)
D	4753 (19.9%)	31330 (60.5%)	4505 (13.0%)
S	5667 (23.7%)	14937 (28.9%)	14732 (42.4%)
Overall	23933 (100%)	51753 (100%)	34767 (100%)

NOTE: In each cell, outside of the parentheses is the number of erroneous segments of a specific score category (row) via a specified aligner (column), and the number in the parentheses is its percentage relative to the overall total. For the definitions of the score categories, see Figure 3.

[a] Specifically, we used E-INS-1 and E-INS-i, respectively, as the progressive and iterative options of MAFFT.

Table S3. Erroneous segments in which reconstructed MSA is “far-apart” from true MSA (via E-INS-1 (*i.e.*, progressive) of MAFFT)

	w/ many block-wise steps [a]	w/ many site-wise steps [b]	long blocks [c]
Score category	MAFFT (E-INS-1)	MAFFT (E-INS-1)	MAFFT (E-INS-1)
12 primates			
I	1.5%	2.6%	1.0%
D	2.2%	4.3%	1.7%
S	0.1%	0.1%	0.1%
Overall	0.7%	1.3%	0.6%
15 mammals			
I	38.7%	37.3%	6.8%
D	35.3%	33.3%	5.8%
S	3.9%	2.9%	0.9%
Overall	26.5%	25.0%	5.7%
9 fast-evolving mammals			
I	71.3%	71.1%	9.2%
D	59.8%	57.4%	6.4%
S	14.2%	12.4%	2.0%
Overall	55.5%	54.5%	8.5%

The same note and footnotes apply as those for Table 3.

Table S4. Frequencies of errors of different types in MSAs among 9 fast-evolving mammals

Error type	MAFFT (E-INS-1)	(%)	MAFFT (E-INS-i)	(%)	Prank (Best-fit)	(%)
Shift	5069	(21.21%)	10035	(19.42%)	11019	(31.77%)
Merge	273	(1.14%)	732	(1.42%)	429	(1.24%)
Purge	686	(2.87%)	1570	(3.04%)	645	(1.86%)
Split	1	(0.004%)	0	(0%)	104	(0.30%)
Ex-nihilo	0	(0%)	0	(0%)	20	(0.06%)
v-Merge [a]	104	(0.44%)	159	(0.31%)	221	(0.64%)
v-Split [b]	5	(0.02%)	6	(0.01%)	62	(0.18%)
CII [c]	47	(0.20%)	420	(0.81%)	15	(0.04%)
iCII [d]	57	(0.24%)	597	(1.16%)	9	(0.03%)
Others [e]	56	(0.23%)	136	(0.26%)	155	(0.45%)
Mixture [f]	1655	(6.92%)	3720	(7.20%)	2841	(8.19%)
(Paired) [g]	(771)	(3.23%)	(1469)	(2.84%)	(1293)	(3.73%)
Complex [h]	15948	(66.73%)	34294	(66.37%)	19161	(55.25%)
Total	23901	(100%)	51669	(100%)	34681	(100%)

The same note and footnotes apply as those for Table 4.

Table S5. Combinations of error types associated with pairs of blocks

A. Via MAFFT (E-INS-i, *i.e.*, iterative)

**Error-type combination [a]**	**12 primates (%)**	**15 mammals (%)**	**9 fast-evolving mammals (%)**
Merge + Shift	4575 (74.2%)	20809 (62.1%)	2947 (57.2%)
Shift + Shift	153 (2.5%)	3417 (10.2%)	699 (13.6%)
v-Merge + Shift	418 (6.8%)	2108 (6.3%)	384 (7.5%)
Merge + Merge	89 (1.4%)	962 (2.9%)	156 (3.0%)
Merge + iCII	45 (0.7%)	865 (2.6%)	102 (2.0%)
p-v-Merge [b] + Shift	253 (4.1%)	602 (1.8%)	71 (1.4%)
iCII + Shift	46 (0.7%)	478 (1.4%)	48 (0.9%)
Merge + Purge	36 (0.6%)	459 (1.4%)	90 (1.7%)
CII + Merge	13 (0.2%)	278 (0.8%)	45 (0.9%)
Purge + Shift	17 (0.3%)	244 (0.7%)	44 (0.9%)
Other combinations	522 (8.5%)	3283 (9.8%)	568 (11.0%)
Total	6167 (100%)	33505 (100%)	5154 (100%)

B. Via Prank (Best-fit)

**Error-type combination [a]**	**12 primates (%)**	**15 mammals (%)**	**9 fast-evolving mammals (%)**
Merge + Shift	1390 (42.6%)	11481 (33.7%)	1191 (27.8%)
Split + Shift	495 (15.2%)	6404 (18.8%)	617 (14.4%)
Shift + Shift	100 (3.1%)	3881 (11.4%)	838 (19.6%)
v-Merge + Shift	188 (5.8%)	2447 (7.2%)	422 (9.9%)
v-Split + Shift	137 (4.2%)	1397 (4.1%)	200 (4.7%)
Merge + Split	160 (4.9%)	1013 (3.0%)	94 (2.2%)
v-Merge + Split	79 (2.4%)	802 (2.4%)	106 (2.5%)
v-Split + Merge	48 (1.5%)	523 (1.5%)	63 (1.5%)
p-v-Merge [b] + Shift	103 (3.2%)	495 (1.5%)	60 (1.4%)
CEII + Shift	59 (1.8%)	392 (1.1%)	39 (0.9%)
Other combinations	502 (15.4%)	5272 (15.5%)	647 (15.1%)
Total	3261 (100%)	34107 (100%)	4277 (100%)

(continues to the next page)

C. Via MAFFT (E-INS-1, *i.e.*, progressive)

Error-type combination [a]	12 primates (%)	15 mammals (%)	9 fast-evolving mammals (%)
Merge + Shift	4551 (68.6%)	18317 (57.2%)	1356 (50.2%)
Shift + Shift	187 (2.8%)	3801 (11.9%)	491 (18.2%)
v-Merge + Shift	570 (8.6%)	2873 (9.0%)	349 (12.9%)
Merge + Merge	92 (1.4%)	759 (2.4%)	68 (2.5%)
p-v-Merge [b] + Shift	290 (4.4%)	551 (1.7%)	21 (0.8%)
Merge + iCII	46 (0.7%)	485 (1.5%)	14 (0.5%)
Merge + Purge	36 (0.5%)	419 (1.3%)	40 (1.5%)
iCII + Shift	76 (1.1%)	366 (1.1%)	16 (0.6%)
v-Split + Shift	32 (0.5%)	312 (1.0%)	20 (0.7%)
v-Merge + Split	60 (0.9%)	273 (0.9%)	16 (0.6%)
Other combinations	692 (10.4%)	3886 (12.1%)	311 (11.5%)
Total	6632 (100%)	32042 (100%)	2702 (100%)

NOTE: The paired types listed here are the top 10 pairs for MSAs of 15 mammals.

[a] The names of error types (except [b]) are the same as those in Table 4.

[b] Partial vertical merge, where gaps (or sequences) of different lengths are vertically aligned.

Table S6. Measures of misestimated indel counts

Measure [a]	ordinary difference		L1 distance		deletion bias	
Score category	MAFFT (E-INS-i)	Prank (Best-fit)	MAFFT (E-INS-i)	Prank (Best-fit)	MAFFT (E-INS-i)	Prank (Best-fit)
12 primates						
I	0.22	0.09	1.16	0.29	0.97	0.02
D	-0.07	0.24	1.18	1.07	0.76	-0.08
S	-0.06	-0.05	0.07	0.07	0.002	-0.001
Overall	-0.05	-0.03	0.38	0.12	0.22	-0.001
15 mammals						
I	0.54	0.07	2.44	1.18	2.12	-0.02
D	-0.28	-3.46	4.24	5.82	3.01	-0.53
S	-0.44	-0.23	0.51	0.54	0.05	0.02
Overall	-0.23	-0.36	2.65	1.14	1.81	-0.04
9 fast-evolving mammals						
I	-0.43	-15.3	3.76	17.5	3.11	-0.41
D	-87.9	-215.9	93.6	218.9	-6.21	-22.5
S	4.24	-0.93	7.00	1.27	4.58	0.000
Overall	-52.1	-35.6	59.2	37.1	-2.12	-3.14

NOTE: The number in each cell is the specified measure of the misestimated indel count by a specified aligner (column), averaged over erroneous segments belonging to a specified score category (row).

[a] Three measures of the indel count misestimation. See section *M8* of *Methods* for their definitions.

Table S7. Measures of misestimated indel counts (via MAFFT, E-INS-1 (*i.e.*, progressive))

Measure [a]	ordinary difference	L1 distance	deletion bias
Score category	MAFFT (E-INS-1)	MAFFT (E-INS-1)	MAFFT (E-INS-1)
12 primates			
I	0.05	0.69	0.53
D	-0.13	1.12	0.69
S	-0.06	0.07	0.003
Overall	-0.06	0.37	0.21
15 mammals			
I	-0.68	3.21	2.18
D	-0.63	3.82	2.59
S	-0.44	0.49	0.04
Overall	-0.59	2.56	1.65
9 fast-evolving mammals			
I	-72.7	75.0	0.38
D	-7.78	10.0	3.46
S	-1.25	1.32	0.15
Overall	-43.2	45.0	0.93

The same note and footnote apply as those for Table S6.

Supplementary figures (with legends)

A. True MSA

```
(position)     0123456789

mamm001        CTTT------
mamm002        CTTT------
mamm003        CTTT------
mamm004        CTTT------
mamm005        CTTT------
mamm006        GTTT------
mamm007        TTTT------
mamm008        CTTT------
mamm009        TGTT------
mamm010        CGTT------
mamm011        CGTT------
mamm012        CGTT---TGT
mamm013        CGTT------
mamm014        -GTTCAC---
mamm015        -TTTCAC---
```

B. Reconstructed MSA

```
(position)     0123456

mamm001        CTTT---
mamm002        CTTT---
mamm003        CTTT---
mamm004        CTTT---
mamm005        CTTT---
mamm006        GTTT---
mamm007        TTTT---
mamm008        CTTT---
mamm009        TGTT---
mamm010        CGTT---
mamm011        CGTT---
mamm012        CGTTTGT
mamm013        CGTT---
mamm014        GTTCA-C
mamm015        TTTCA-C
```

C. Position-shift map

(position)	0	1	2	3	4	5	6
mamm001	0	0	0	0	-	-	-
mamm002	0	0	0	0	-	-	-
mamm003	0	0	0	0	-	-	-
mamm004	0	0	0	0	-	-	-
mamm005	0	0	0	0	-	-	-
mamm006	0	0	0	0	-	-	-
mamm007	0	0	0	0	-	-	-
mamm008	0	0	0	0	-	-	-
mamm009	0	0	0	0	-	-	-
mamm010	0	0	0	0	-	-	-
mamm011	0	0	0	0	-	-	-
mamm012	0	0	0	0	-3	-3	-3
mamm013	0	0	0	0	-	-	-
mamm014	-1	-1	-1	-1	-1	-	0
mamm015	-1	-1	-1	-1	-1	-	0

D. Partitioning into position-shift blocks

(position)	0	1	2	3	4	5	6
mamm001	0	0	0	0	-	-	-
mamm002	0	0	0	0	-	-	-
mamm003	0	0	0	0	-	-	-
mamm004	0	0	0	0	-	-	-
mamm005	0	0	0	0	-	-	-
mamm006	0	0	0	0	-	-	-
mamm007	0	0	0	0	-	-	-
mamm008	0	0	0	0	-	-	-
mamm009	0	0	0	0	-	-	-
mamm010	0	0	0	0	-	-	-
mamm011	0	0	0	0	-	-	-
mamm012	0	0	0	0	-3	-3	-3
mamm013	0	0	0	0	-	-	-
mamm014	-1	-1	-1	-1	-1	-	0
mamm015	-1	-1	-1	-1	-1	-	0

Figure S1. Erroneous segment caused by two interacting errors.

The true MSA was simulated along the tree in Figure 2 B. The red and blue blocks (with shifts -1 and -3, respectively,) were paired and associated with a "merge + iCII." See the legend of Figure 1 for details on the notation.

A. True MSA

```
(position)      00000000001111
                01234567890123

mamm001         AGTT--AAATCC-G
mamm002         AGTT--AAATCC-G
mamm003         AGTT--AAATCC-G
mamm004         AGTT--AAATCC-G
mamm005         AGTT--AAATCC-G
mamm006         AGTT--AAGTCC-G
mamm007         AGTT--AAGTCC-G
mamm008         AGTT--AAGTCC-G
mamm009         TGGT--GACTCAAG
mamm010         AGGT--AACTCC-G
mamm011         AGTA--AATGCG-G
mamm012         AGTA--AACGCC-G
mamm013         AGTA--CGCTCG--
mamm014         TGTTTAAAGTTC-T
mamm015         ATTTTACCCTCC-C
```

B. Reconstructed MSA

```
(position)      0000000000111
                0123456789012

mamm001         --AGTTAAATCCG
mamm002         --AGTTAAATCCG
mamm003         --AGTTAAATCCG
mamm004         --AGTTAAATCCG
mamm005         --AGTTAAATCCG
mamm006         --AGTTAAGTCCG
mamm007         --AGTTAAGTCCG
mamm008         --AGTTAAGTCCG
mamm009         -TGGTGACTCAAG
mamm010         --AGGTAACTCCG
mamm011         --AGTAAATGCGG
mamm012         --AGTAAACGCCG
mamm013         ---AGTACGCTCG
mamm014         TGTTTAAAGTTCT
mamm015         ATTTTACCCTCCC
```

C. Position-shift map

(position)	0	1	2	3	4	5	6	7	8	9	10	11	12
mamm001	-	-	2	2	2	2	0	0	0	0	0	0	-1
mamm002	-	-	2	2	2	2	0	0	0	0	0	0	-1
mamm003	-	-	2	2	2	2	0	0	0	0	0	0	-1
mamm004	-	-	2	2	2	2	0	0	0	0	0	0	-1
mamm005	-	-	2	2	2	2	0	0	0	0	0	0	-1
mamm006	-	-	2	2	2	2	0	0	0	0	0	0	-1
mamm007	-	-	2	2	2	2	0	0	0	0	0	0	-1
mamm008	-	-	2	2	2	2	0	0	0	0	0	0	-1
mamm009	-	1	1	1	1	-1	-1	-1	-1	-1	-1	-1	-1
mamm010	-	-	2	2	2	2	0	0	0	0	0	0	-1
mamm011	-	-	2	2	2	2	0	0	0	0	0	0	-1
mamm012	-	-	2	2	2	2	0	0	0	0	0	0	-1
mamm013	-	-	-	3	3	3	3	1	1	1	1	1	1
mamm014	0	0	0	0	0	0	0	0	0	0	0	0	-1
mamm015	0	0	0	0	0	0	0	0	0	0	0	0	-1

D. Partitioning into position-shift blocks

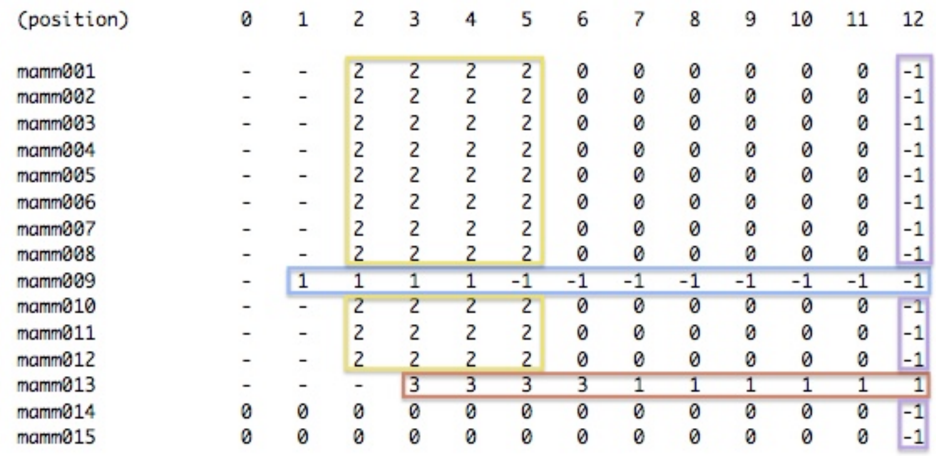

Figure S2. Erroneous segment caused by three interacting errors.

The true MSA was simulated along the tree in Figure 2 B. The current prototype script judged that this erroneous segment contains complex errors. Manually, however, the errors can be interpreted as “shift + iCII + shift,” which were caused, roughly speaking, by the yellow block (with shift 2), the blue composite block (with shifts 1 and -1), and the red composite block (with shifts 3 and 1), respectively. And the purple block (with shift -1) is considered as accompanying the blue composite block. See the legend of Figure 1 for details on the notation.

A. True MSA

```
(position)    01234567

mamm001       CA--GG-A
mamm002       CA--GG-A
mamm003       GA--GG-A
mamm004       GA--GG-A
mamm005       GA--GG-A
mamm006       CA--GG-A
mamm007       CA--GG-A
mamm008       CA--GG-A
mamm009       CA--GG-A
mamm010       CA--GG-A
mamm011       -A--GG-A
mamm012       CA--GGGA
mamm013       CA--GG-G
mamm014       CAGTAG-C
mamm015       CA--GG-C
```

B. Reconstructed MSA

```
(position)    01234567

mamm001       CAG--GA-
mamm002       CAG--GA-
mamm003       GAG--GA-
mamm004       GAG--GA-
mamm005       GAG--GA-
mamm006       CAG--GA-
mamm007       CAG--GA-
mamm008       CAG--GA-
mamm009       CAG--GA-
mamm010       CAG--GA-
mamm011       A----GGA
mamm012       CAG--GGA
mamm013       CAG--GG-
mamm014       CAGTAGC-
mamm015       CAG--GC-
```

C. Position-shift map

(position)	0	1	2	3	4	5	6	7
mamm001	0	0	-2	-	-	0	-1	-
mamm002	0	0	-2	-	-	0	-1	-
mamm003	0	0	-2	-	-	0	-1	-
mamm004	0	0	-2	-	-	0	-1	-
mamm005	0	0	-2	-	-	0	-1	-
mamm006	0	0	-2	-	-	0	-1	-
mamm007	0	0	-2	-	-	0	-1	-
mamm008	0	0	-2	-	-	0	-1	-
mamm009	0	0	-2	-	-	0	-1	-
mamm010	0	0	-2	-	-	0	-1	-
mamm011	-1	-	-	-	-	1	1	0
mamm012	0	0	-2	-	-	0	0	0
mamm013	0	0	-2	-	-	0	-1	-
mamm014	0	0	0	0	0	0	-1	-
mamm015	0	0	-2	-	-	0	-1	-

D. Partitioning into position-shift blocks

(position)	0	1	2	3	4	5	6	7
mamm001	0	0	-2	-	-	0	-1	-
mamm002	0	0	-2	-	-	0	-1	-
mamm003	0	0	-2	-	-	0	-1	-
mamm004	0	0	-2	-	-	0	-1	-
mamm005	0	0	-2	-	-	0	-1	-
mamm006	0	0	-2	-	-	0	-1	-
mamm007	0	0	-2	-	-	0	-1	-
mamm008	0	0	-2	-	-	0	-1	-
mamm009	0	0	-2	-	-	0	-1	-
mamm010	0	0	-2	-	-	0	-1	-
mamm011	-1	-	-	-	-	1	1	0
mamm012	0	0	-2	-	-	0	0	0
mamm013	0	0	-2	-	-	0	-1	-
mamm014	0	0	0	0	0	0	-1	-
mamm015	0	0	-2	-	-	0	-1	-

Figure S3. Erroneous segment caused by four interacting errors.

The true MSA was simulated along the tree in Figure 2 B. The current prototype script judged that this erroneous segment contains complex errors. Manually, however, the errors can be interpreted as “vertical split + shift + merge + shift,” which were caused by the green block (with shift -1), the blue one (with shift 1), the red one (with shift -1) and the yellow one (with shift -2), respectively. See the legend of Figure 1 for details on the notation.

A. 12 primates via MAFFT (E-INS-i)

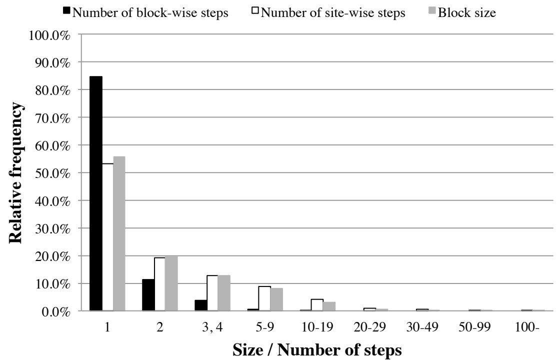

B. 12 primates via Prank

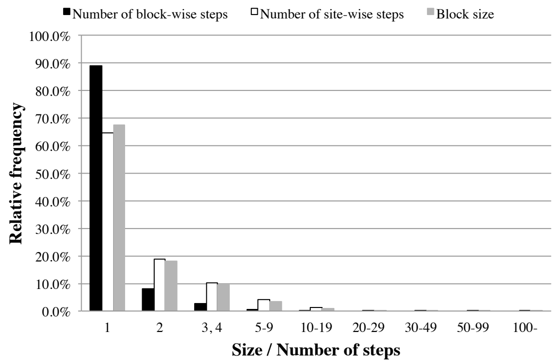

C. 15 mammals via MAFFT (E-INS-i)

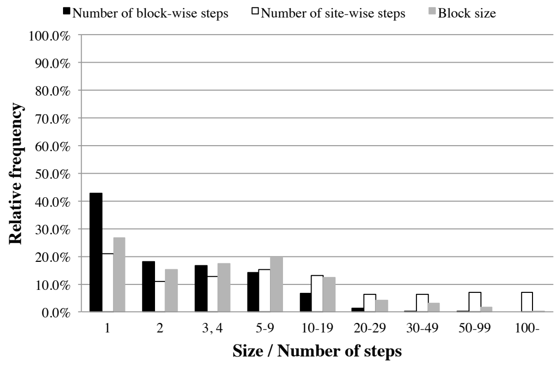

D. 15 mammals via Prank

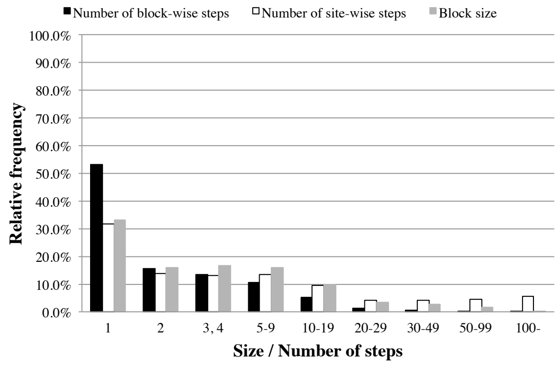

E. 9 FE mammals via MAFFT (E-INS-i)

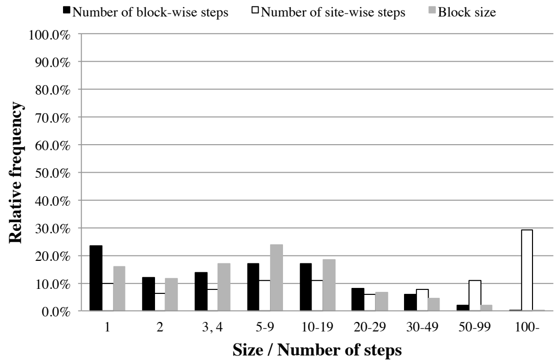

F. 9 FE mammals via Prank

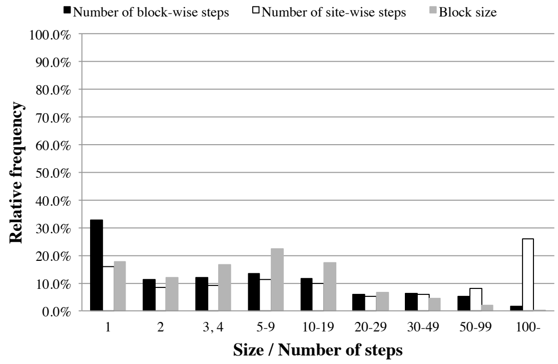

Figure S4. Distribution of "separation"s between true and reconstructed MSAs. **(A,C,E)** Via MAFFT, E-INS-i (*i.e.*, iterative). **(B,D,F)** Via Prank. **(A,B)** With 12 primates. **(C,D)** With 15 mammals. **(E,F)** With 9 fast-evolving (FE) mammals. The black and white columns are used for the numbers of block-wise steps and of site-wise steps, respectively, in each erroneous segment. The grey columns are used for the size of each position-shift block. The abscissa represents the class of the values of each "separation" measure. The ordinate represents the relative frequency (in percent).

A. 12 primates via MAFFT (E-INS-1)

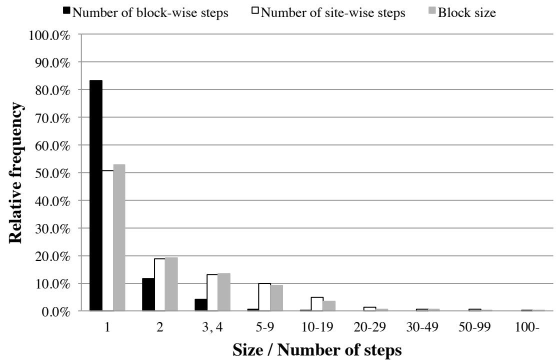

B. 15 mammals via MAFFT (E-INS-1)

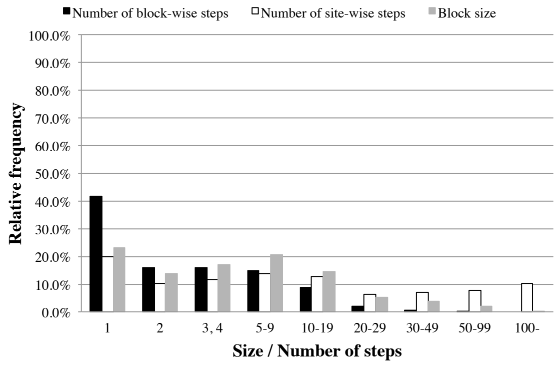

C. 9 FE mammals via MAFFT (E-INS-1)

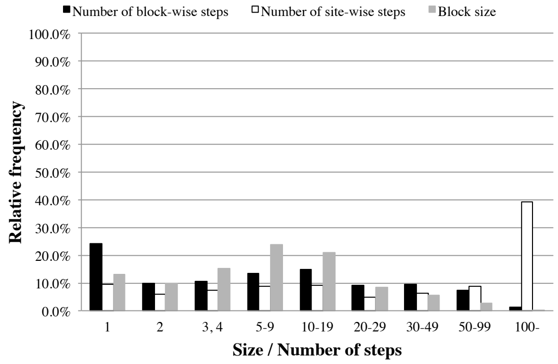

Figure S5. Distribution of "separation"s between true and reconstructed MSAs (via MAFFT, E-INS-1 (*i.e.*, progressive)).

(A) With 12 primates. **(B)** With 15 mammals. **(C)** With 9 fast-evolving (FE) mammals. See the legend of Figure S4 for the notation and the convention.

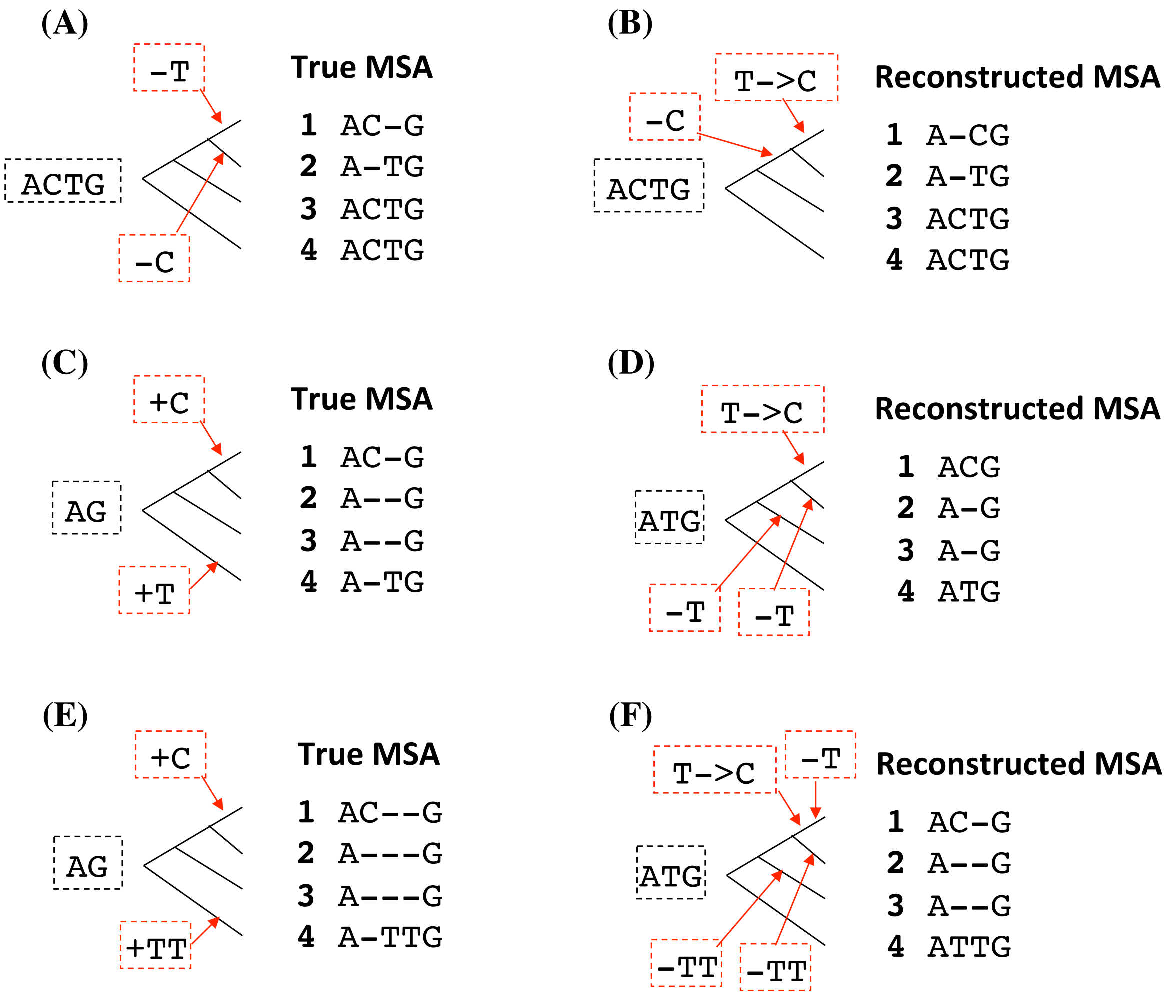

Figure S6. Schematic illustrations of some MSA-specific error types.

(A, B) A "vertical merge" of two deletion-induced gaps. **(C, D)** A "collapse of independent insertions (CII)." **(E, F)** An "incomplete collapse of independent insertions (iCII)." Each of panels **(A)**, **(C)** and **(E)** depicts the true evolutionary history (on the left) and the resulting true MSA (on the right). Each of panels **(B)**, **(D)**, and **(F)** depicts the reconstructed MSA (on the right) and one of its parsimonious evolutionary interpretations (on the left). In each panel, the numbers 1-4 in bold face represent the aligned sequences and their corresponding external nodes in the tree. The most recent common ancestor of the aligned sequences is in the black dashed box at the root. In red dashed boxes, "+X," "-Y," and "Z->W" represent the insertion of subsequence X, deletion of subsequence Y, and the substitution from residue Z to residue W, respectively.

A. 12 primates via MAFFT (E-INS-1)

(X = L1 distance; Y = deletion bias)

Y \ X	0	1	2	3	4,5	6–10	11–20	21–	Total
...,–21	0	0	0	0	0	0	0	1	1
–20,...,–11	0	0	0	0	0	0	0	2	2
–10,...,–6	0	0	0	0	0	2	0	0	2
–5,–4	0	0	0	0	9	0	1	0	10
–3	0	0	0	52	0	0	1	0	53
–2	0	0	656	13	3	0	0	0	672
–1	0	4559	119	52	0	1	0	0	4731
0	117173	2831	2879	34	14	0	0	0	122931
1	0	6576	146	60	4	0	1	0	6787
2	0	0	3723	28	4	0	0	0	3755
3	0	0	0	2314	3	0	0	0	2317
4,5	0	0	0	0	2291	1	0	0	2292
6,...,10	0	0	0	0	0	867	0	0	867
11,...,20	0	0	0	0	0	0	2	0	2
21,...	0	0	0	0	0	0	0	0	0
Total	117173	13966	7523	2553	2328	871	5	3	144422

B. 15 mammals via MAFFT (E-INS-1)

(X = L1 distance; Y = deletion bias)

Y \ X	0	1	2	3	4,5	6–10	11–20	21–	Total
...,–21	0	0	0	0	0	0	0	113	113
–20,...,–11	0	0	0	0	0	0	0	41	41
–10,...,–6	0	0	0	0	0	59	20	30	109
–5,–4	0	0	0	0	547	135	18	17	717
–3	0	0	0	1346	228	121	15	4	1714
–2	0	0	3242	108	730	280	15	5	4380
–1	0	17925	323	2873	675	214	23	7	22040
0	132008	748	16334	268	1679	409	21	9	151476
1	0	24738	487	3371	840	270	36	10	29752
2	0	0	14306	321	1203	418	17	4	16269
3	0	0	0	13754	620	238	25	13	14650
4,5	0	0	0	0	29222	557	32	18	29829
6,...,10	0	0	0	0	0	30322	83	43	30448
11,...,20	0	0	0	0	0	0	6823	45	6868
21,...	0	0	0	0	0	0	0	517	517
Total	132008	43411	34692	22041	35744	33023	7128	876	308923

C. 9 FE mammals via MAFFT (E-INS-1)

(X = L1 distance; Y = deletion bias)

Y \ X	0	1	2	3	4,5	6–10	11–20	21–	Total
...,–21	0	0	0	0	0	0	0	59	59
–20,...,–11	0	0	0	0	0	0	0	18	18
–10,...,–6	0	0	0	0	0	19	16	26	61
–5,–4	0	0	0	0	46	50	49	16	161
–3	0	0	0	87	45	66	17	8	223
–2	0	0	269	52	101	109	35	13	579
–1	0	959	71	266	127	114	57	12	1606
0	5765	129	1360	87	249	159	59	13	7821
1	0	1289	131	267	166	159	74	25	2111
2	0	0	788	112	185	186	81	21	1373
3	0	0	0	736	169	167	94	26	1192
4,5	0	0	0	0	1681	387	206	52	2326
6,...,10	0	0	0	0	0	2689	537	172	3398
11,...,20	0	0	0	0	0	0	1933	402	2335
21,...	0	0	0	0	0	0	0	976	976
Total	5765	2377	2619	1607	2769	4105	3158	1839	24239

Figure S7. Different features of indel count misestimations by MAFFT (progressive).

The figure shows the results via MAFFT, E-INS-1 (*i.e.*, progressive). **(A)** With 12 primates. **(B)** With 15 mammals. **(C)** With 9 fast-evolving (FE) mammals. The panels follow the same notation and convention as those in Figure 6.
